# Analytical Solution to Assess the Induced Seismicity Potential of Faults in Pressurized and Depleted Reservoirs

**DOI:** 10.1029/2020JB020436

**Published:** 2021-01-19

**Authors:** Haiqing Wu, Victor Vilarrasa, Silvia De Simone, Maarten Saaltink, Francesco Parisio

**Affiliations:** ^1^ Department of Civil and Environmental Engineering (DECA) Universitat Politécnica de Catalunya (UPC) Barcelona Spain; ^2^ Associated Unit: Hydrogeology Group (UPC‐CSIC) Barcelona Spain; ^3^ Institute of Environmental Assessment and Water Research (IDAEA) Spanish National Research Council (CSIC) Barcelona Spain; ^4^ Mediterranean Institute for Advanced Studies (IMEDEA) Spanish National Research Council (CSIC) Esporles Spain; ^5^ Univ Rennes CNRS Géosciences Rennes‐UMR 6118 Rennes France; ^6^ Chair of Soil Mechanics and Foundation Engineering Institute of Geotechnics Technische Universität Bergakademie Freiberg Freiberg Germany

**Keywords:** reservoir pressurization/depletion, fault stability, inclusion theory, induced earthquakes, permeable and impermeable faults

## Abstract

Displaced faults crossing the reservoir could significantly increase the induced earthquake frequency in geo‐energy projects. Understanding and predicting the stress variation in such cases is essential to minimize the risk of induced seismicity. Here, we adopt the inclusion theory to develop an analytical solution for the stress response to pore pressure variations within the reservoir for both permeable and impermeable faults with offset ranging from zero to the reservoir thickness. By analyzing fault stability changes due to reservoir pressurization/depletion under different scenarios, we find that (1) the induced seismicity potential of impermeable faults is always larger than that of permeable faults under any initial and injection conditions—the maximum size of the fault undergoing failure is 3–5 times larger for impermeable than for permeable faults; (2) stress concentration at the corners results in the occurrence of reversed slip in normal faults with a normal faulting stress regime; (3) while fault offset has no impact on the slip potential for impermeable faults, the slip potential increases with the offset for permeable faults, which indicates that non‐displaced permeable faults constitute a safer choice for site selection; (4) an impermeable fault would rupture at a lower deviatoric stress, and at a smaller pressure buildup than a permeable one; and (5) the induced seismicity potential is overestimated and the injectivity underestimated if the stress arching (i.e., the poromechanical coupling) is neglected. This analytical solution is a useful tool for site selection and for supporting decision making during the lifetime of geo‐energy projects.

## Key Points

- We propose an analytical solution for the stress response to pore pressure changes in the reservoir crossed by a fault with an arbitrary dip
- Induced seismicity potential of impermeable faults is always larger than that of permeable faults under any initial and injection conditions
- Induced seismicity potential is overestimated and the injectivity underestimated if the poromechanical coupling is neglected

## 1 Introduction

Induced seismicity has become a widespread issue as a result of the proliferation of geo‐energy projects (Foulger et al., 2018). On one hand, geothermal energy production and geologic carbon storage are essential technologies to reach zero or negative net carbon emissions. On the other hand, the increased energy demand is boosting other operations, such as seasonal natural gas storage, subsurface energy storage, and disposal of wastewater from conventional and non‐conventional oil and gas production. Injecting or pumping fluids at depth—a widespread practice in geo‐energy operations—alters the in‐situ stress field and may lead to fault rupture and induced seismicity (Buijze et al., 2017; Ellsworth, 2013; Grigoli et al., 2018). In several cases, the authorities have decided to cancel projects believed to be associated with large induced earthquakes and a non‐exhaustive list includes the Deep Heat Mining Project in Basel, Switzerland (Deichmann & Giardini, 2009; Haring et al, 2008; Terakawa et al., 2012), the Castor Natural Storage Project, Spain (Del Potro & Diez 2015; Juanes et al., 2017; Villaseñor et al. 2020), and the Enhanced Geothermal System (EGS) Project at Pohang, South Korea (Ellsworth et al., 2019; Grigoli et al., 2018; Lee et al., 2019). To reduce the risks of induced seismicity and safely promote sustainable energy development, tools to predict and subsequently mitigate induced seismicity should be developed.

Despite considerable advancements in understanding the triggering mechanisms of induced seismicity in recent years, forecast and mitigation of induced seismicity remains challenging and some fundamental questions remain open (Elsworth et al., 2016; Lee et al., 2019). Potential triggering mechanisms include pore pressure diffusion caused by single phase (Shapiro & Dinske, 2009; Simpson et al., 1988) and multiphase flows (Zbinden et al., 2017), poroelastic and thermally induced stress changes (Chang & Segall, 2016; De Simone et al., 2017; Langenbruch & Zoback, 2016), and strength weakening due to geochemical reactions (Rohmer et al., 2016; Vilarrasa et al., 2019). These mechanisms, separately or acting jointly, can lead to fault slip (Lehner, 2019; Lele et al., 2016; Orlic et al., 2013; Orlic & Wassing, 2013; Rutqvist et al., 2016; Van den Bogert, 2015; Van Wees et al., 2017) and nucleation of dynamic rupture (Buijze et al., 2017, 2019; Galis et al., 2017, 2019; Garagash & Germanovich, 2012) on different geological settings (Bourne & Oates, 2017; Haug et al., 2018), even at very large distances (Goebel et al., 2017).

Faults intersecting the injection/pumping formation undergo pore pressure and stress changes, affecting their stability. Pore pressure changes are controlled by the hydraulic properties of faults, which are highly variable, ranging from conductive faults to flow barriers (Caine et al., 1996). For example, low‐permeable faults are present at the Snohvit CO_2_ storage site, Norway (Chiaramonte et al., 2013; Hansen et al., 2013), at Pohang EGS project, South Korea (Ellsworth et al., 2019; Kim et al., 2018), and at many compartmentalized reservoirs (e.g., Castelletto et al., 2013), while permeable faults are found at the Groningen gas field, the Netherlands (Jansen et al., 2019; Van Wees et al., 2014) and the Corinth rift, Greece (Duverger et al., 2015; Geraud et al., 2006). Stress changes arise when the reservoir deformation is restricted, as in the case of closed or compartmentalized reservoirs, and they are governed by the poromechanical properties of the rock—the stiffer the rock, the larger the induced stress—and by the fault offset, which generates an additional stress concentration (Buijze et al., 2017; Galis et al., 2017, 2019). Such generated stress could lead to an increase in induced earthquake frequency, as was observed in the Groningen gas field (NAM, 2016; Van Wees et al., 2014, 2017).

Numerical simulations can account for great physical and geometrical complexity, but the computational cost often prevents systematic explorations of the parametric space. Analytical methods offer an alternative to obtain fast estimations, but require more stringent hypotheses and simplifications on the geometry and physics of the problem when compared with numerical methods. Interestingly, their drawback turns into an advantage when the perspective is changed and the goal becomes a quick and efficient parametric space analysis, ultimately highlighting the factors controlling the problem. For the problem of reservoir pressurization/depletion, Eshelby's inclusion theory (Eshelby, 1957) is at the heart of several analytical solutions describing displacement, strain, and stress fields in an infinite half‐space with an elliptic inclusion (Segall, 1985, 1992; Segall & Fitzgerald, 1998). The theory was applied to study subsidence and induced seismicity (Segall, 1985, 1989; Segall et al., 1994), recognizing the influence of reservoir geometry and orientation (Soltanzadeh & Hawkes, 2008, 2009) and the importance of including the contribution of crack‐tip resistance to fault strength (Wang et al., 2016). Existing analytical solutions either assume non‐displaced faults (Segall, 1985, 1992; Segall & Fitzgerald, 1998; Soltanzadeh & Hawkes, 2008, 2009; Wang et al., 2016) or displaced but permeable faults (Jansen et al., 2019). No solution currently exists for low‐permeable faults that cross the reservoir with an offset: the aim of our contribution is to fill this knowledge gap and analyze the difference in terms of fault stability between permeable and low‐permeable faults crossing a pressurized/depleted reservoir for both non‐displaced and displaced faults.

In this paper, we propose an analytical solution for stress variations in response to injection/pumping into a reservoir crossed by a fault that could be either permeable or impermeable with offset ranging from zero to the reservoir thickness. Note that by stress variations we refer to the total stress changes, whereas for the effective stress, we explicitly mention effective in our terminology. The structure of the paper is as follows. In Section 2, we introduce the conceptual problem, develop the analytical solution, and show its validation. In Section 3, we present the methods to assess fault stability and fault slip potential based on our solution for both permeable and impermeable faults. In Section 4, we illustrate the effect of fault permeability on fault stability and perform systematical parametric space analyses of fault offset, fault dip, initial stress state, and pressure change. Finally, we provide extended discussion of the results and its principal implications in Section 5.

## 2 Analytical Solution for Stress Changes Around a Fault Crossing a Pressurized/Depleted Reservoir

### 2.1 Problem Formulation and Assumptions

**Figure 1:**
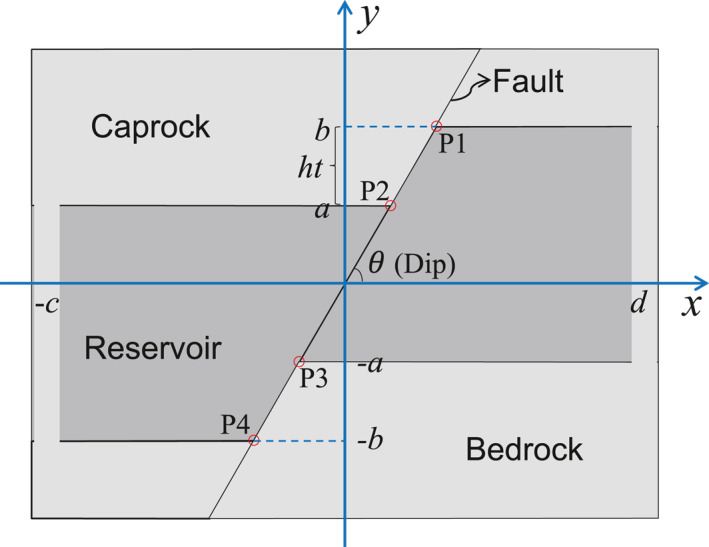
Geological model and its schematic geometry. Pore pressure changes within the reservoir on one or both sides of the fault depending on whether the fault is impermeable or permeable, respectively. The caprock and bedrock are assumed impermeable and thus, no pressure changes occur outside the reservoir.

We evaluate the induced stress arising in a deep reservoir crossed by a displaced permeable or impermeable fault as a consequence of fluid injection or production. The reservoir is treated as an inclusion that is hydraulically disconnected from the overlying caprock and underlying bedrock (Figure 1). A displaced fault with an arbitrary dip angle θ crosses the whole reservoir and extends to the surrounding rock, dividing the entire domain into two parts: the left part is the hanging wall and the right part is the footwall for a normal fault (Jansen et al., 2019). A non‐displaced fault is a particular case in which the fault offset is zero. The fault offset, *ht*, is defined as *b*–*a* (see Figure 1), and fault geometry is parameterized by four corner points (P1, P2, P3, and P4). The height (thickness) and width of the faulted reservoir are *a* + *b* and *c* + *d*, respectively, where the width can be assumed as infinite by imposing *c* = *d* = ∞. The reservoir length is assumed as infinite in the out‐of‐plane direction.

We adopt the solid mechanics sign convention of stress and strain, that is, negative normal components denote compression, and a positive shear stress is assumed to rotate the material element in the counterclockwise direction, which indicates that the left part of the fault moves downward relative to the right part. For pore pressure, a negative pore pressure change refers to production and a positive one to injection.

We apply the following hypotheses: (1) the reservoir, assumed horizontal, elastic, homogeneous, and isotropic, and its surroundings have identical stiffness but different permeability and porosity—the latter implies that flow and pressure changes take place exclusively within the reservoir; (2) two‐dimensional (2D) plane strain conditions apply based on the assumption that the reservoir extends infinitely in the out‐of‐plane direction; (3) quasi‐steady‐state uniform pore pressure changes occur in the reservoir as a consequence of injection/production, that is, the transient effect of flow is neglected; and (4) reservoir depth is great enough so that the effect of the free surface can be neglected (Lehner, 2019).

### 2.2 Analytical Solution

According to the inclusion theory (Eshelby, 1957; Mura, 1987; Rudnicki, 2011), pore pressure changes induce stress variation *σ*_*ij*_ in the reservoir as (see Appendix A for the full mathematical development)

(1) $$\sigma_{ij}\left(x,y\right)=C\left[\iint_{\Omega }g_{ij}\left(x,y,\varsigma ,\xi \right)d\Omega -\pi \delta_{ij}\delta_{\Omega }\right]=C\left[G_{ij}\left(x,y\right)-\pi \delta_{ij}\delta_{\Omega }\right],$$

(2) $$C=\frac{\left(1-2\nu \right)\alpha \Delta p}{2\pi \left(1-\nu \right)},$$

where Ω is the inclusion domain, *g*_*ij*_ and *G*_*ij*_ represent the Green's function for stress and its surface integral, respectively, *x* and *y* are the Cartesian coordinates, *ς* and *ξ* denote the coordinate values within the domain Ω, *α* is the Biot's coefficient, *ν* is the Poisson's ratio, and Δ*p* is the pore pressure change. *δ*_*ij*_ is the Kronecker delta, which equals 1 if *i* = *j* or 0 if *i* ≠ *j* and *δ*_Ω_ is the modified Kronecker delta, which equals 1 if (x,y)∈Ω or 0 if (x,y)∉Ω.

Green's function *g*_ij_ gives the magnitude of the stress in the *i*‐th direction at point (*x*, *y*) in response to a body force in the *j*‐th direction applied at point (*ς*, *ξ*; see Equations A17–A19). To perform their integration over the inclusion domain as in Equation 1, we set the origin to coincide with the midpoint of the fault (Figure 1). The integration domain is different, whether we are in the case of permeable or impermeable faults. In the former case, pore pressure changes within the reservoir on both sides of the fault. For the latter case, pore pressure changes only on the side of the fault where injection or depletion takes place. In the permeable case, the entire inclusion consists of two trapezoids (Figure 1), each of which can be divided into two subdomains to simplify the integration. Thus, we apply the superposition principle of integral to combine the solutions for rectangular and triangular domains, which returns (see Appendix B for the full derivation) as

(3) $$\begin{array}{c} G_{xx}\left(x,y\right)=-G_{yy}\left(x,y\right)=a\tan\frac{y+b}{x+c}-a\tan\frac{y-a}{x+c}+a\tan\frac{y-b}{x-d}-a\tan\frac{y+a}{x-d}\\ -\left[f_{1}\left(x,y,-b\right)-f_{1}\left(x,y,a\right)+f_{1}\left(x,y,b\right)-f_{1}\left(x,y,-a\right)\right]{\sin\;}^{2}\;\theta \\ -\frac{\sin\;\theta \;\cos\;\theta }{2}\ln\left(\frac{f_{2}\left(x,y,-b\right)f_{2}\left(x,y,b\right)}{f_{2}\left(x,y,a\right)f_{2}\left(x,y,-a\right)}\right)-\pi \delta_{\Omega }, \end{array}$$

(4) $$\begin{array}{ccc} G_{xy}\left(x,y\right) & = & \left[f_{1}\left(x,y,-b\right)-f_{1}\left(x,y,a\right)+f_{1}\left(x,y,b\right)-f_{1}\left(x,y,-a\right)\right]\sin\;\theta \;\cos\;\theta \\ & & -\frac{{\sin\;}^{2}\;\theta }{2}\ln\frac{f_{2}\left(x,y,-b\right)f_{2}\left(x,y,b\right)}{f_{2}\left(x,y,a\right)f_{2}\left(x,y,-a\right)}+\frac{1}{2}\ln\frac{f_{3}\left(x+c,y+b\right)f_{3}\left(x-d,y-b\right)}{f_{3}\left(x+c,y-a\right)f_{3}\left(x-d,y+a\right)}, \end{array}$$

where the functions *f*_1_, *f*_2_, and *f*_3_ are

(5) $$f_{1}\left(x,y,\hat{y}\right)=a\tan\frac{\left(x-\hat{y}\cot\;\theta \right)\cot\;\theta +\left(y-\hat{y}\right)}{x-y\;\cot\;\theta },$$

(6) $$f_{2}\left(x,y,\hat{y}\right)={\left(x-\hat{y}\cot\;\theta \right)}^{2}+{\left(y-\hat{y}\right)}^{2},$$

(7) $$f_{3}\left(x-\hat{x},y-\hat{y}\right)={\left(x-\hat{x}\right)}^{2}+{\left(y-\hat{y}\right)}^{2},$$

and where *a, b, c,* and *d* are the geometrical parameters shown in Figure 1. The last term $\left(-\pi \delta_{\Omega }\right)$ in Equation 3 results from the solution of improper integral (Courant & John, 1989) because the Green's function for stress becomes unbounded for points (*x*, *y*) located in the inclusion domain. The corners of two trapezoidal domains are singularities for the solutions (Equations 3 and 4; see Appendix B). The vertical fault is a special case of inclined fault, which is obtained by setting θ= 90° (Equations B21–B22). And faults with no offset are also a special case in which *a = b* (Equations B23–B24).

When substituting Equations 3 and 4 into Equation 1, we obtain the *x*–*y* planar solution for describing the distribution of induced stress in the pressurized or depleted reservoir and its surrounding rock. Our solution is consistent to the one developed by Jansen et al. (2019), for the case of a horizontal infinite reservoir crossed by a permeable fault. However, our current solution is also valid for any arbitrary reservoir width, with the solution for the infinite reservoir being a special case, that is, *c = d = ∞*.

Fault stability and its likelihood of rupture depend on the distribution of the normal and tangential stress components along the fault plane. Thus, the above *x*–*y* planar solution along the fault plane needs to be transformed into the coordinate system placed on the fault and oriented along it. We apply the stress transformation with axis rotation (Equations C4 and C5) to derive the closed expressions for such induced stress along an arbitrary fault plane with dip angle θ, which yields (see Appendix C for the full derivation)

(8) $$\begin{array}{c} {\bar{\sigma }}_{n}\left(y\;\cot\;\theta ,y\right)=-\frac{\pi }{2}\left[\mathrm{sgn}\left(y+b\right)-\mathrm{sgn}\left(y-a\right)+\mathrm{sgn}\left(y-b\right)-\mathrm{sgn}\left(y+a\right)\right]{\sin\;}^{2}\;\theta \\ -\cos\;2\;\theta (a\tan\frac{y+b}{y\;\cot\;\theta +c}-a\tan\frac{y-a}{y\;\cot\;\theta +c}+a\tan\frac{y-b}{y\;\cot\;\theta -d}\\ -a\tan\frac{y+a}{y\;\cot\;\theta -d}-\pi \delta_{\Omega })+\frac{\sin\;2\;\theta }{4}\ln\frac{f_{4}\left(y,b\right)}{f_{4}\left(y,a\right)}\\ -\frac{\sin\;2\;\theta }{2}\ln\frac{f_{3}\left(y\;\cot\;\theta +c,y+b\right)f_{3}\left(y\;\cot\;\theta -d,y-b\right)}{f_{3}\left(y\;\cot\;\theta +c,y-a\right)f_{3}\left(y\;\cot\;\theta -d,y+a\right)}-\pi \delta_{\Omega }, \end{array}$$

(9) $$\begin{array}{c} \bar{\tau }\left(y\;\cot\;\theta ,y\right)=-\frac{\pi }{4}\left[\mathrm{sgn}\left(y+b\right)-\mathrm{sgn}\left(y-a\right)+\mathrm{sgn}\left(y-b\right)-\mathrm{sgn}\left(y+a\right)\right]\sin\;2\;\theta \\ +\sin\;2\;\theta (a\tan\frac{y+b}{y\;\cot\;\theta +c}-a\tan\frac{y-a}{y\;\cot\;\theta +c}+a\tan\frac{y-b}{y\;\cot\;\theta -d}-a\tan\frac{y+a}{y\;\cot\;\theta -d}-\pi \delta_{\Omega })-\frac{{\sin\;}^{2}\;\theta }{2}\ln\frac{f_{4}\left(y,b\right)}{f_{4}\left(y,a\right)}\\ -\frac{\cos\;2\;\theta }{2}\ln\frac{f_{3}\left(y\;\cot\;\theta +c,y+b\right)f_{3}\left(y\;\cot\;\theta -d,y-b\right)}{f_{3}\left(y\;\cot\;\theta +c,y-a\right)f_{3}\left(y\;\cot\;\theta -d,y+a\right)}, \end{array}$$

where $\sigma_{n}\left(y\cot\theta ,\;y\right)\;\text{and}\;\tau \left(y\cot\theta ,\;y\right)$ are the induced normal and tangential stress components along the fault plane, respectively; they will be shortened to $\sigma_{n}\;\text{and}\;\tau$ for convenience hereafter. The stress components with an overbar denote the dimensionless stress components, which are normalized by the scaling parameter *C* (Equation 2). The sgn (*•*) is the sign function defined as 1 if (*•*) > 0, 0 if (*•*) = 0 or −1 if (*•*) < 0, and function *f*_4_ is defined as

(10) $$f_{4}\left(y,\hat{y}\right)={\left(y+\hat{y}\right)}^{2}{\left(y-\hat{y}\right)}^{2}.$$

The four corners, P1, P2, P3, and P4, on the fault plane are singularities of Equations 8 and 9 (Figure 1). With such a general solution, one can easily find the solutions for the special cases of vertical faults (Equations C8 and C9) and zero offset faults (Equations C10 and C11). In particular, $\sigma_{n}\;\text{and}\;\tau$ just correspond to $\sigma_{xx}\;\text{and}\;\sigma_{xy}$ for the case of vertical faults, respectively. In the above equations, the segment P1–P2 of the fault belongs to the inclusion, while the segment P3–P4 belongs to the surroundings (see Appendix C).

For an impermeable fault, we assume that the pore pressure change is restricted to the side of the fault where injection/production takes place, while pore pressure on the other side remains unaltered. Thus, the integration of the Green's function for stress only entails one part of the inclusion domain, that is, one trapezoidal domain. Considering that fluid is injected into the left‐hand side of the domain, such integrations are

(11) $$\begin{array}{c} G_{xx}\left(x,y\right)=-G_{yy}\left(x,y\right)\\ =a\tan\frac{y+b}{x+c}-a\tan\frac{y-a}{x+c}-\frac{\sin\;\theta \;\cos\;\theta }{2}\ln\left(\frac{f_{2}\left(x,y,-b\right)}{f_{2}\left(x,y,a\right)}\right)\\ -\left[f_{1}\left(x,y,-b\right)-f_{1}\left(x,y,a\right)\right]{\sin\;}^{2}\;\theta -\pi \delta_{\Omega }, \end{array}$$

(12) $$\begin{array}{c} G_{xy}\left(x,y\right)=\left[f_{1}\left(x,y,-b\right)-f_{1}\left(x,y,a\right)\right]\sin\;\theta \;\cos\;\theta \\ -\frac{{\sin\;}^{2}\;\theta }{2}\ln\frac{f_{2}\left(x,y,-b\right)}{f_{2}\left(x,y,a\right)}+\frac{1}{2}\ln\frac{f_{3}\left(x+c,y+b\right)}{f_{3}\left(x+c,y-a\right)}, \end{array}$$

and the dimensionless induced normal and tangential stress components along the fault plane are

(13) $$\begin{array}{ccc} {\bar{\sigma }}_{n}\left(y\;\cot\;\theta ,y\right) & = & -\cos\;2\;\theta \left(a\tan\frac{y+b}{y\;\cot\;\theta +c}-a\tan\frac{y-a}{y\;\cot\;\theta +c}-\pi \delta_{\Omega }\right)\\ & & -\frac{\pi }{2}\left[\mathrm{sgn}\left(y+b\right)-\mathrm{sgn}\left(y-a\right)\right]{\sin\;}^{2}\;\theta \\ & & +\frac{\sin\;2\;\theta }{4}\ln\frac{{\left(y+b\right)}^{2}}{{\left(y-a\right)}^{2}}\\ & & -\frac{\sin\;2\;\theta }{2}\ln\frac{f_{3}\left(y\;\cot\;\theta +c,y+b\right)}{f_{3}\left(y\;\cot\;\theta +c,y-a\right)}-\pi \delta_{\Omega }, \end{array}$$

(14) $$\begin{array}{ccc} \bar{\tau }\left(y\;\cot\;\theta ,y\right) & = & \sin\;2\;\theta \left(a\tan\frac{y+b}{y\;\cot\;\theta +c}-a\tan\frac{y-a}{y\;\cot\;\theta +c}-\pi \delta_{\Omega }\right)\\ & & -\frac{\sin^{2}\;\theta }{2}\ln\frac{{\left(y+b\right)}^{2}}{{\left(y-a\right)}^{2}}-\frac{\pi }{4}\left[\mathrm{sgn}\left(y+b\right)-\mathrm{sgn}\left(y-a\right)\right]\sin\;2\;\theta \\ & & -\frac{\cos\;2\;\theta }{2}\ln\frac{f_{3}\left(y\;\cot\;\theta +c,y+b\right)}{f_{3}\left(y\;\cot\;\theta +c,y-a\right)}. \end{array}$$

The corners of the left‐hand trapezoidal domains of the fault are singularities of Equations 11 and 12, and the corners P2 and P4 on the fault plane are singularities of Equations 13 and 14 (Figure 1). The whole impermeable fault belongs to the surroundings for fluid injection into the left‐hand side of the fault because we apply the right limit, that is, the limit that the argument approaches the fault from its right‐hand side, as the value of the fault plane. Note that in Equation 11 the extra term originating from improper integral is always $-\pi \delta_{\Omega }$ because it depends only on the integrand (the limit of integration at improper points only depends on the integrand) and not on the geometry. The solution is similar in the case of injection into the right‐hand side of the fault. To avoid confusion or repetitions, in the following we will always consider the case of injection into the left‐hand side as an example to represent the impermeable case.

### 2.3 Validation Against Numerical Solution

To verify the accuracy and correctness of our analytical solution, we compare fluid injection‐induced stress distribution along a permeable and an impermeable fault against numerical solutions. The numerical simulations are performed with the fully coupled finite element code CODE_BRIGHT (Olivella et al., 1994, 1996). The geometry is shown in Figure 1. We adopt dimensions and rock properties as in Jansen et al. (2019) in order to also compare our results with theirs (Table 1). In the numerical simulations, we mimic the impermeable rock, that is, caprock and bedrock, and the impermeable fault by assigning low values of intrinsic permeability, that is, 10^−18^ m^2^. We impose mechanical boundary conditions of zero normal displacement to the lateral and lower boundaries and an overburden of −70 MPa on the upper boundary, corresponding to a depth of 3.5 km. We assume that the initial stress state is isotropic. The magnitude of the initial stress and pressure is irrelevant because we are interested in the stress changes induced by pore pressure changes and both the hydraulic and mechanical processes are linear. We impose a pressure buildup of 20 MPa in the reservoir, which is the entire reservoir in the case of permeable fault while half of it is in the case of impermeable fault. We make sure that the size of the reservoir is large enough to minimize the boundary effects.

**Table 1:** Geometrical Parameters of the Reservoir and Rock Properties Adopted for the Validation Example

Parameter	Physical meaning	Value	Unit
*a*	Geometrical parameters (refer to Figure 1)	100	m
*b*		200	m
*c*		2,000	m
*d*		2,000	m
θ	Fault dip	60	°
μ	Shear modulus	6,500	MPa
ν	Poisson's ratio	0.15	–
α	Biot's coefficient	0.9	–
Δ*p*	Pressure buildup	20	MPa

**Figure 2:**
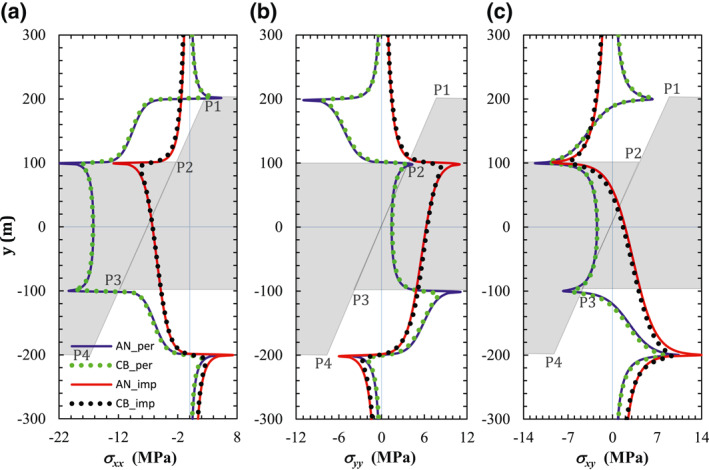
Comparison between analytically and numerically evaluated induced (a) horizontal, (b) vertical, and (c) *x–y* planar shear stress components along the fault plane. The results along the fault are projected on the vertical axes *y*. The legend is shown in (a), indicating that solid lines represent the analytical results (AN) and dotted lines represent the numerical results with CODE_BRIGHT (CB), for both a permeable (per) and an impermeable (imp) fault. A schematic of the reservoir geometry, with the four corners, is indicated by the gray background.

We compare the numerical and analytical results for a permeable and an impermeable fault (Figure 2). Further results are presented in Section S1. The numerically computed induced stress on the fault plane is almost identical to the analytical one. Small discrepancies near the corners are a consequence of the existence of singularities for the analytical solution, which leads to an infinite stress, and of the discrete nature of the numerical solution. We also consider the case of vertical permeable fault crossing a horizontal infinite reservoir in order to compare our results with those of Jansen et al. (2019). Comparisons are shown in Section S2 and they exhibit good agreement.

## 3 Induced Seismicity Potential Assessment

### 3.1 Coulomb Failure Stress and Coulomb Failure Stress Change

We assess the fault stability by means of the Coulomb Failure Stress (*CFS*; King, 1994)

(15) $$CFS=\left|\tau +\tau^{0}\right|+\eta_{\text{st}}\left(\sigma_{n}^{\prime }+\sigma_{n}^{\prime 0}\right),$$

where $\eta_{\text{st}}$ is the static friction coefficient, superscript 0 represents the initial state, and superscript ′ denotes effective stress. Here, the initial normal and tangential stress components on the fault plane are also calculated according to the stress transformation (Equations C4 and C5). Shear stress always drives the fault to slip regardless of whether it is positive or negative. The second term on the right‐hand side of Equation 15 denotes the fault slip resistance and increases as the effective normal stress becomes more compressive. A positive value of *CFS* indicates that slip is activated in the direction of the shear stress along the fault, that is, a positive shear stress represents the normal slip and a negative shear stress represents the reversed slip for a normal fault (Jha & Juanes, 2014).

For the assessment of induced seismicity and to identify whether a portion of the fault becomes more or less stable, we use the *CFS* change (ΔCFS) as

(16) $$\Delta CFS=\left|\tau +\tau^{0}\right|-\left|\tau^{0}\right|+\eta_{\text{st}}\sigma_{n}^{\prime },$$

A positive ΔCFS implies that the induced stress is driving the fault toward failure and eventually co‐seismic slip. *CFS* andΔCFS can be normalized by the scaling parameter *C* (Equation 2) as

(17) $${CFS}_{D}=\left|\bar{\tau }+\frac{\tau^{0}}{C}\right|+\eta_{\text{st}}\left({\bar{\sigma }}_{n}+\frac{\alpha \Delta p}{C}+\frac{\sigma_{n}^{0}+\alpha p^{0}}{C}\right),$$

(18) $$\Delta {CFS}_{D}=\left|\frac{\bar{\tau }+\tau^{0}}{C}\right|-\left|\frac{\tau^{0}}{C}\right|+\eta_{\text{st}}\left({\bar{\sigma }}_{n}+\frac{\alpha \Delta p}{C}\right),$$

where we make use of Equations 8 and 9 for permeable faults or Equations 13 and 14 for impermeable faults, and the subscript D denotes dimensionless variables. Here the dimensionless effective pore pressure change, that is, the term αΔp/C, is an initial physical property within the reservoir, which is independent of the process of integration. Thus, the segment P1–P4 undergoes the same pore pressure change as the reservoir for permeable faults, and the segment P2–P4 undergoes the same pore pressure change as the left‐reservoir compartment for fluid injection into the left‐hand side of the impermeable fault.

### 3.2 Fault Slip Size

To quantitatively evaluate the fault slip potential, and thus, the induced seismicity potential, we define the fault slip size as

(19) $$S_{i}=\frac{\ell_{i}}{\sin\;\theta },$$

where $\ell_{i}$ is a continuous interval in coordinate *y* with *CFS* > 0. The slipping area can be discontinuous, so more than one *S*_*i*_ may exist. We assume that the greatest magnitude of induced earthquakes is proportional to the maximum fault slip size, defined as

(20) $$S_{\max}=\max\left(S_{i}\right),$$

which can be expressed in dimensionless form as

(21) $$S_{D\max}=\frac{S_{\max}}{L_{s}}=\max\frac{\left(\ell_{i}\right)}{\left(a+b\right)},$$

where *L*_s_ is a characteristic length of fault, here assumed as the length of the fault intercepting the reservoir

(22) $$L_{s}=\frac{a+b}{\sin\;\theta },$$

Assuming that each grain is restricted by its surrounding grains, that is, the existence of cohesion between grains, the fault will not slide until the maximum unstable patch reaches a threshold. We set *S*_Dmax_ = 0.01 as the threshold of fault slip in this paper, that is, the fault is always regarded as stable for *S*_Dmax_ < 0.01.

### 3.3 Properties of the Base Case Scenario

We evaluate the stress variation and fault stability as well as the fault slip potential for a pressurized reservoir whose properties are derived from laboratory measurements on Berea sandstone (Makhnenko & Labuzet 2015; Vilarrasa et al., 2016; Table 2). We assume the same geometrical model as in Section 2.3 (Figure 1 and Table 1), with the center of the reservoir at 3.5 km depth, and the initial stress state (normal faulting stress regime) as shown in Table 2.

**Table 2:** Properties of Berea Sandstone and the Initial Stress State of the Reservoir Adopted for the Failure Potential Analysis

Parameter	Physical meaning	Value	Unit
θ	Fault dip	60	°
*ht*_D_	Dimensionless fault offset	1/3	–
μ	Shear modulus	4,600	MPa
ν	Poisson's ratio	0.29	–
α	Biot's coefficient	0.7	–
Δ*p*_D_	Dimensionless pressure buildup	4/7	–
*p* ^0^	Initial pore pressure	35	MPa
$\sigma_{yy}^{0}$	Initial vertical stress	−70	MPa
$k_{0}$	Stress ratio of horizontal to vertical stress	0.6	–
$\sigma_{xy}^{0}$	Initial shear stress in the *x–y* plane	0	MPa
$\eta_{\text{st}}$	Static friction coefficient	0.6	–
*C*	Scaling parameter for stress (Equation 2)	1.318	MPa
$CFS_{D}^{0}$	Initial dimensionless *CFS* (Equation 17)	−1.954	–

To generalize the problem, we normalize the coordinate *y* and the fault offset by the reservoir thickness, and we scale the pressure buildup by the initial pore pressure, which yields the dimensionless variables

(23) $$y_{D}=\frac{y}{\left(a+b\right)},$$

(24) $$ht_{D}=\frac{\left(b-a\right)}{\left(a+b\right)},$$

(25) $$\Delta p_{D}=\frac{\Delta p}{p^{0}}.$$

We compare the results for the two scenarios of permeable and impermeable faults to understand the influence of the hydraulic properties of faults on the fault stability and fault slip potential. In addition to these base case scenarios, we perform a parametric space analyses to explore the effects of fault geometry, initial stress state, and operational aspects. We compare in all cases the difference between permeable and impermeable faults.

## 4 Results

### 4.1 Effect of Fault Permeability in the Base Case Scenario

**Figure 3:**
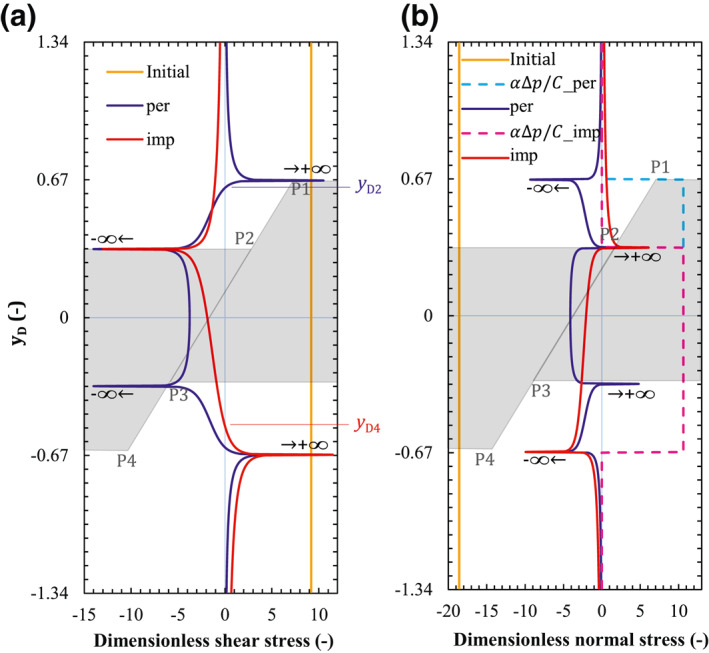
Dimensionless (a) shear and (b) normal stress components on the fault plane for the case of permeable (blue lines) and impermeable (red lines) fault. Dashed lines are the term of pressure buildup normalized by the scaling parameter *C*. Blue auxiliary line and label denote the zero point of $\bar{\tau }$ for a permeable fault, and the red ones for an impermeable fault. The results along the fault are projected on the vertical dimensionless axes *y*_*D*_. A schematic of the reservoir geometry, with the four corners, is indicated by the gray background.

We evaluate the dimensionless induced shear and normal stress components on the fault plane according to Equations 8 and 9 for a permeable fault as well as Equations 13 and 14 for an impermeable fault (Figure 3). For the permeable fault, the dimensionless induced stress is symmetrical with respect to *y*_D_ = 0, as it is reflected by Equations 8 and 9 when the geometric parameters are *c* = *d*. The corner points are singular, such that the induced shear stress tends to + ∞ at P1 and P4, and − ∞ at P2 and P3 (for representation purposes, the infinite shear stress is cut off to a finite value). The induced normal stress has a reverse behavior with respect to the induced shear stress (compare Figures 3a and 3b), that is, it tends to −∞ at P1 and P4, and + ∞ at P2 and P3. The entire fault plane except for a tiny vicinity at corners P2 and P3 shows a negative induced normal stress implying an increase in slip resistance, which contributes to the fault stability.

Unlike the permeable case, the induced shear and normal stress components are not symmetrical with respect to *y*_D_ = 0 for the impermeable fault, but the feature of reverse behavior for shear and normal stress components still holds (compare Figures 3a and 3b). The induced shear and normal stress components tend to infinity at corners P2 and P4 as a consequence of injecting from the left‐hand side. These stress singularities in both the permeable and impermeable faults correspond to the points of stress concentration. The infinite value is a theoretical consequence of the integration of the Green's function and it is unrealistic for faults in nature where the material will undergo nonlinear deformation bounding stress values. We also plot the initial shear and effective normal stress components as well as the pressure buildup in Figure 3 to identify the contribution of each term to fault slip.

**Figure 4:**
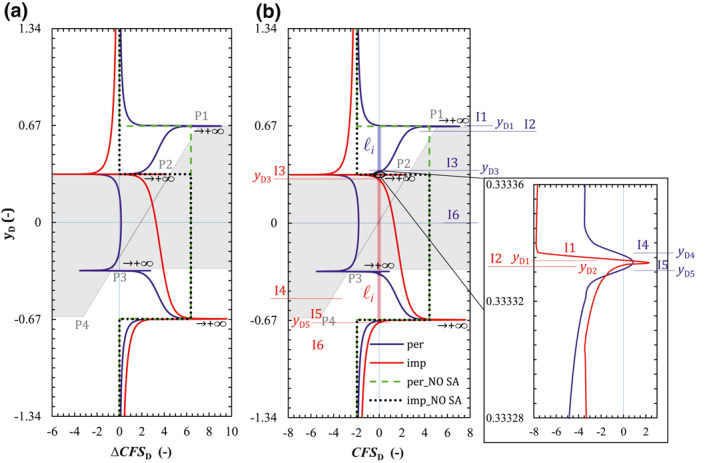
Dimensionless (a) Coulomb Failure Stress Change ($\Delta CFS_{D}$) and (b) Coulomb Failure Stress (*CFS*_D_) on the fault plane for the case of permeable (blue lines) and impermeable (red lines) faults. I*i* and *y*_D*i*_ are the subinterval and zero point of *CFS*_D_, respectively, in which the blue auxiliary lines and labels denote the ones for a permeable fault, and the red ones for an impermeable fault. The results along the fault are projected on the vertical dimensionless axes *y*_*D*_. A schematic of the reservoir geometry, with the four corners, is indicated by the gray background. We also show the case of no stress arching (denoted by NO SA), that is, stress changes induced by poromechanical effects are neglected. The legend is shown in (b).

The dimensionless Δ*CFS* ($\Delta {CFS}_{D},\;$ Equation (18) along the fault plane, reflecting variations in the fault stability, remains symmetrical with respect to *y*_D_ = 0 for the permeable fault (Figure 4a) because the arithmetic operations of the symmetrical stress does not alter its symmetry. The stability of the permeable fault decreases everywhere, except for a small region close to the internal corners P2 and P3. Around the external corners, $\;\Delta {CFS}_{D}$ reaches its maximum value because of the stress concentration, which will likely induce fault slip locally. Conversely, the impermeable fault (Figure 4a) becomes more stable above the internal corner P2 and less stable below it. To determine the actual fault stability and assess whether failure conditions occur, *CFS*_D_ is computed as ${CFS}_{D}^{0}$ (Table 2) plus $\Delta {CFS}_{D}$. It results that *CFS*_D_ has the same trend as $\Delta {CFS}_{D}$ (compare Figures 4a and 4b), but shifted by the magnitude of ${CFS}_{D}^{0}$. Therefore, the size of the fault that potentially undergoes failure $\left({CFS}_{D}>0\right)\;$ is smaller than that where stability decreases $\left(\Delta {CFS}_{D}>0\right)$.

To analyze the slip mechanism of permeable faults, given the symmetry of *CFS*_D_, we divide the upper half part of the *CFS*_D_ curve into six subintervals (I1, I2, I3, I4, I5, and I6) by the four zero‐points of *CFS*_D_ (*y*_D1_, *y*_D3_, *y*_D4_, and *y*_D5_) and the zero point of $\bar{\tau }$ (*y*_D2_, see Figure 3a; blue symbols in Figures 3a and 4b). For impermeable faults, the whole *CFS*_D_ curve, however, is divided into six subintervals (I1, I2, I3, I4, I5, and I6) by the four zero points of *CFS*_D_ (*y*_D1_, *y*_D2_, *y*_D3,_ and *y*_D5_) and the zero point of $\bar{\tau }$ (*y*_D4_, see Figure 3a; red symbols in Figures 3a and 4b). Detailed fault state and mechanics for each subinterval are shown in Table 3, in which both the shear and normal stress components changes belong to the fluid injection‐induced poroelastic response. Overall, pore pressure buildup, which mainly results in the decrease of slip resistance, induces fault slip in the reservoir or makes it less stable. The poroelastic response, however, represents a stabilizing effect on the fault within the reservoir except for a small vicinity around the corners because of the local stress concentration. While the poroelastic response has a small negative effect on fault stability both in the caprock and bedrock for permeable faults, it performs a positive effect on fault stability in the caprock but a negative effect in the bedrock for impermeable faults (Figure 4a).

**Table 3:** Slip Mechanism of Permeable and Impermeable Faults in the Base Case Scenario

Fault	Subinterval	State (see Figure 4b)	Mechanism (see Figure 3)
Permeable	I1	Less stable	Increase in shear stress
	I2	Normal slip	Increase both in shear stress and pore pressure
	I3	Normal slip	Increase in pore pressure
	I4	Stable	Decrease in shear stress
	I5	Reversed slip	Reversed increase in shear stress
	I6	Stable	Decrease in shear stress and increase in normal stress
Impermeable	I1	More stable	Decrease in shear stress
	I2	Reversed slip	Reversed increase in shear stress
	I3	Stable	Decrease in shear stress
	I4	Normal slip	Increase in pore pressure
	I5	Normal slip	Increase both in shear stress and pore pressure
	I6	Less stable	Increase in shear stress

Following such combined characteristics of pore pressure buildup and poroelastic response, a permeable fault has four disconnected unstable patches (two normal slip patches and two reversed slip patches, Figure 4b), and an impermeable one has two unstable patches (one normal slip patch and one reversed slip patch, Figure 4b). The unstable patches of permeable faults, located between the external and internal corners (i.e., between P1 and P2 and between P3 and P4), are symmetric with respect to *y*_D_ = 0 and are separated by the stable central portion of the reservoir (between the internal corners P2 and P3). In contrast, practically the whole section of impermeable faults located in the pressurized reservoir become unstable. In this case, while *S*_Dmax_ = 0.32 for the permeable fault, it reaches 0.99 for the impermeable case, so both of them slide but the slip size of the impermeable fault is more than three times that of the permeable fault. Multiplying *S*_Dmax_ by the fault characteristic length (Equation 22) yields the dimensional maximum fault slip size, which is *S*_max_ = 110.85 m for the permeable case and *S*_max_ = 342.95 m for the impermeable case.

For illustrative purposes, we include in Figure 4 the case in which the stress arching (Rudnicki 2002; Segall, 1985; Soltanzadeh & Hawkes, 2008) is neglected, that is, stress changes both inside and outside the reservoir induced by poromechanical effects are neglected and the effective normal stress variation equals the pressure changes. Thus, only the pressure buildup in the reservoir induces the increase in *CFS*, that is, $\Delta CFS=\eta_{\text{st}}\alpha \Delta p=8.4\;\text{MPa}$ in the base case scenario, which is significantly larger than the ΔCFS for the case of including the stress arching, except for the infinite values at the corner points. Neglecting stress changes significantly overestimates the decrease in fault stability, because the compression induced in the rock in response to reservoir expansion caused by pressurization is not taken into account. We will discuss this further in Section 5.

### 4.2 Effect of Fault Offset and Fault Dip

**Figure 5:**
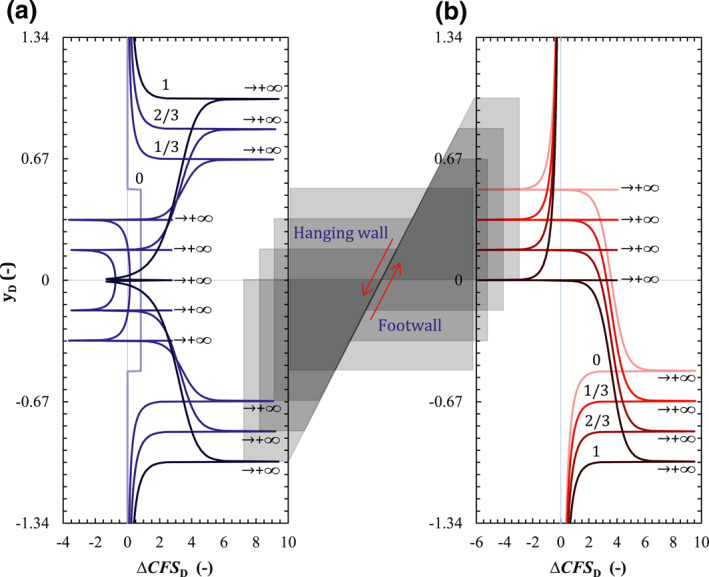
Dimensionless Coulomb Failure Stress Change ($\Delta CFS_{D}$) on the fault plane for several dimensionless fault offsets for (a) permeable and (b) impermeable faults. The numbers on the curves denote the dimensionless fault offset. The results along the fault are projected on the vertical dimensionless axes *y*_*D*_. The gray background indicates the position of the hanging and foot walls, which move simultaneously as the offset increases.

Fault offset affects differently permeable and impermeable faults (Figure 5). While fault stability significantly varies with offset for permeable faults (Figure 5a), impermeable faults undergo the same stability changes, but shifted, coinciding with the center of the pressurized/depleted reservoir (Figure 5b). For a permeable fault, $\Delta {CFS}_{D}$ slightly increases (stability decreases) within the reservoir and it is barely altered in the surrounding rock when the offset is equal to zero (Figure 5a). The stability‐decreasing section increases proportionally to the fault offset and it concentrates at the external corners, where it tends to infinity. The section of the fault where the reservoir is juxtaposed on both sides of the fault presents a slight increase in stability. The size of this stabilized section decreases with fault offset, becoming negligible when the fault offset equals the reservoir thickness. In contrast, the size of the symmetric stability‐decreasing sections between the internal and external corners of the reservoir increases with fault offset. Furthermore, both the stability of the caprock and the bedrock also slightly decreases. For an impermeable fault, the size of the stability‐decreasing section, which is mainly constrained by the reservoir thickness, is independent of fault offset (Figure 5b), because the effect of stress concentration, which is controlled by the horizontal boundaries of the reservoir and the fault plane, is always the same for the impermeable fault regardless of its offset.

**Figure 6:**
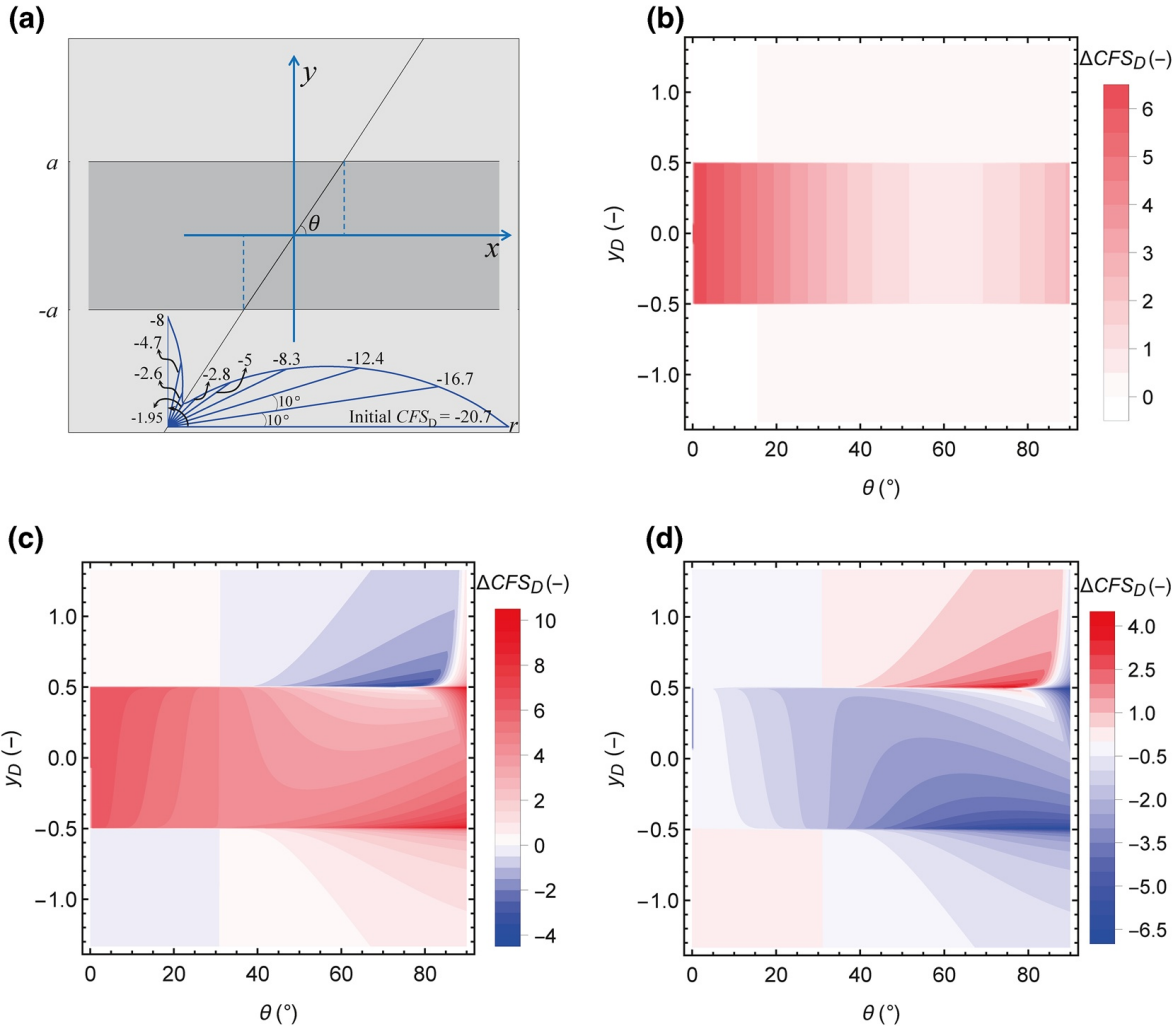
Fault stability changes along the fault plane as a function of the dip angle, θ, for the case of zero‐offset fault. (a) Schematic geometry and $CFS_{D}^{0}$ (shown in a polar coordinate system, the polar angle and diameter denote the fault dip angle and $CFS_{D}^{0}$, respectively), (b)$\;\Delta CFS_{D}$ for the permeable fault, (c)$\;\Delta CFS_{D}$ for the impermeable fault (for comparison purposes, (b) and (c) have the same color scale but the range of the legend is adapted to the values shown in each case), and (d) the difference between $\Delta CFS_{D}$ for the permeable and impermeable faults (i.e., (b–c)), where negative values indicate that the impermeable fault is less stable.

**Figure 7:**
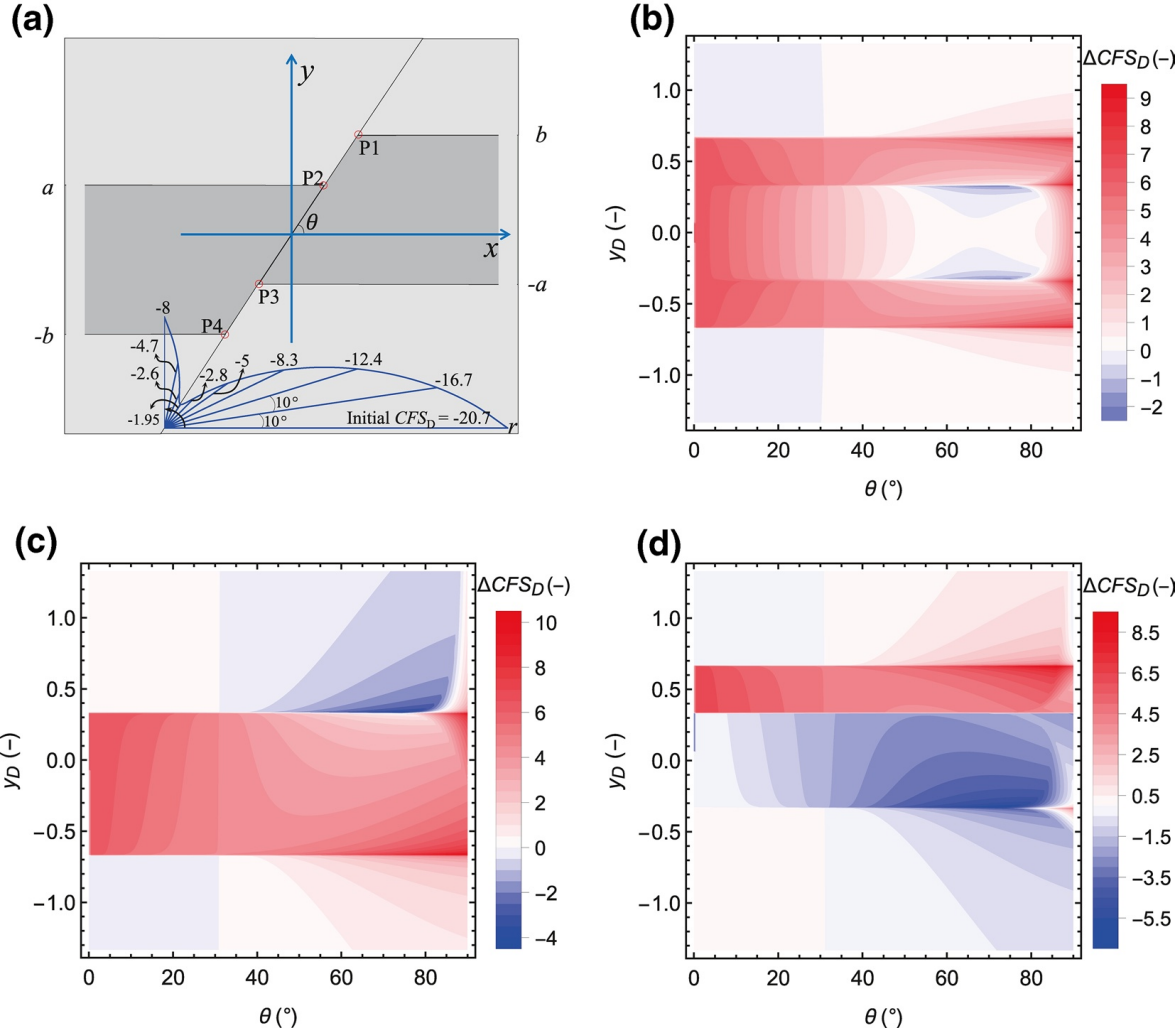
Fault stability changes along the fault plane as a function of the dip angle, θ, for the case with fault dimensionless offset equal to 1/3. (a) Schematic geometry and $CFS_{D}^{0}$ (shown in a polar coordinate system, the polar angle and diameter denote the fault dip angle and $CFS_{D}^{0}$, respectively), (b)$\;\Delta CFS_{D}$ for the permeable fault, (c)$\;\Delta CFS_{D}$ for the impermeable fault (for comparison purposes, (b) and (c) have the same color scale but the range of the legend is adapted to the values shown in each case), and (d) the difference between $\Delta CFS_{D}$ for the permeable and impermeable faults (i.e., (b)–(c)), where negative values indicate that the impermeable fault is less stable.

We analyze the impact of the fault dip, *θ*, (between 0°, horizontal fault, and 90°, vertical fault) on fault stability for both permeable and impermeable faults with no offset (Figure 6) and 1/3 of dimensionless offset (Figure 7). Figure 6a displays the schematic geometric model of no offset fault and the initial *CFS*_D_ as a function of the dip angle. The initial *CFS*_D_ shows that the fault is stable, with the most critical dip around 61°, as expected for a normal faulting stress regime with a fault friction coefficient of 0.6. As a result of reservoir pressurization, fault stability changes differ depending on the hydraulic nature of the fault. For a permeable fault, $\Delta {CFS}_{D}$ is constant in the reservoir for each value of the dip angle (Figure 6b). It exhibits the maximum value for *θ* = 0° (horizontal fault) and the minimum value for *θ* ≈ 61°, while it is close to zero in the surrounding rock for any value of the dip angle. For an impermeable fault, $\Delta {CFS}_{D}\;$is not constant along the fault plane (see also Figure 5b), although its variation is only strongly relevant for θ > 45° (Figure 6c). Its maximum value is always located at the external corners (the horizontal boundary between the reservoir and its surrounding rock) because of the stress concentration (recall Figure 4a), especially for a high dip angle because the effect of stress concentration becomes maximum for the angle of corners at 90° (Ahmadi et al., 2012). $\Delta {CFS}_{D}$ in the caprock decreases for increasing dip angle except for θ > 85°, being negative (more stable) in the range of 31° < *θ* < 85°. $\Delta CFS_{D}$ increases with the dip angle in the bedrock and is positive (less stable) for *θ* > 31°. Note that to assess fault stability, $\Delta {CFS}_{D}$ for either a permeable or an impermeable fault has to be added to the initial *CFS*_D_, which also changes with the dip angle (Figure 6a). The difference in $\Delta {CFS}_{D}$ between the permeable and impermeable faults is negative throughout the reservoir and baserock for almost all the dip angles, that is, the impermeable fault is less stable, and positive over a small area located in the overlying caprock as a consequence of the left‐hand side fluid injection (Figure 6d). A right‐hand side injection would yield symmetrical results, with a positive difference located in the bedrock. Overall, an impermeable fault is more likely to be reactive than a permeable one when there is no offset.

As for the case of 1/3 of dimensionless offset (Figure 7), ${CFS}_{D}^{0}$ is the same as for the fault with no offset (compare Figures 6a and 7a). $\Delta {CFS}_{D}$ for a permeable fault is not constant along its plane and is symmetrical with respect to *y*_D_ = 0 (Figure 7b). The fault has greater stability in the section between the internal corners P2 and P3, where the reservoir is juxtaposed on both sides of the fault, for 50° < *θ* < 80° and lower stability for other dips. For an impermeable fault (Figure 7c), $\Delta {CFS}_{D}$ has the same distribution as for the case of no offset (Figure 6c), with the discontinuity shifted downward as a consequence of the downward shift of the boundary between the reservoir and the surrounding rock (recall Figure 5b). The difference in $\Delta {CFS}_{D}$ between the permeable and impermeable faults shows that for the section above the internal corner P2, the permeable fault is more unstable than the impermeable one, but the impermeable fault is less stable in the rest—a similar result to the zero‐offset case (Figure 7d).

**Figure 8:**
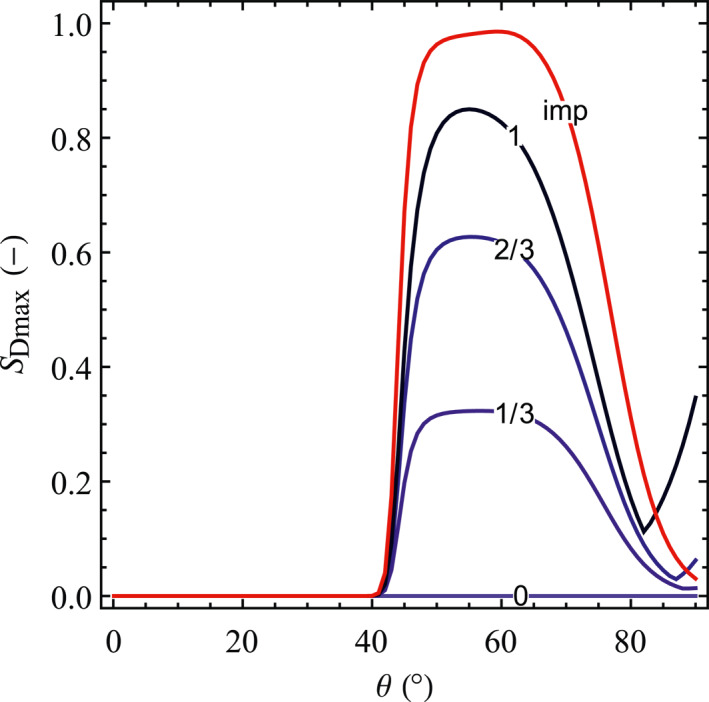
Dimensionless maximum fault slip size (*S*_Dmax_) as a function of fault dip for both permeable (blue color‐scale lines) and impermeable faults (red line) and for several values of the dimensionless offset. The number on the blue lines denotes the dimensionless offset of permeable fault, and the results of different offsets for impermeable fault coincide in one line.

We present the fault slip potential as a function of the fault dip for several fault offsets while keeping the other parameters as the base case scenario, expressed in terms of the dimensionless maximum fault slip size (*S*_Dmax_), in Figure 8. For permeable faults, there is a clear onset value for fault dip (*θ*_o_ = 42°) corresponding to the threshold of fault slip, that is, the fault undergoes slip for dip angles above the onset dip. Once *θ* > *θ*_o_, *S*_Dmax_ increases rapidly and then gradually reaches its peak around *θ*_c_ = 56°, that is, close to the critical dip angle for a normal faulting stress regime. The general trend of *S*_Dmax_ as well as the onset and critical fault dips for slip are almost independent of fault offset, but its maximum value (at *θ* = *θ*_c_) increases with fault offset. According to our initial and injection conditions (Table 2), rupture does not occur when the offset is zero. However, for more critical initial stress state or larger pressure buildup, failure would occur also in the case of zero offset as a consequence of pore pressure buildup (recall Figure 3). When the fault is close to be vertically oriented, there is an inflection point in *S*_Dmax_ that is barely visible for a small‐offset (1/3) fault, but becomes more evident with larger offset. The occurrence of this inflection reflects that *S*_Dmax_ for reversed slip exceeds the one for normal slip, that is, the reversed slip becomes the primary slip form. For impermeable faults, the effect of fault dip on *S*_Dmax_ is similar to the case of permeable fault, with an onset dip *θ*_o_ = 41.3° and a critical dip *θ*_c_ = 59.4°, but no inflection is observed and the maximum value of *S*_Dmax_ is larger, approaching the reservoir thickness at the critical dip. The fault slip potential in impermeable faults is independent of the fault offset also because the effect of stress concentration is always the same, regardless of its offset—a result similar to the fault stability (recall Figure 5b).

### 4.3 Effect of Initial Stress and Pore Pressure Changes

**Figure 9:**
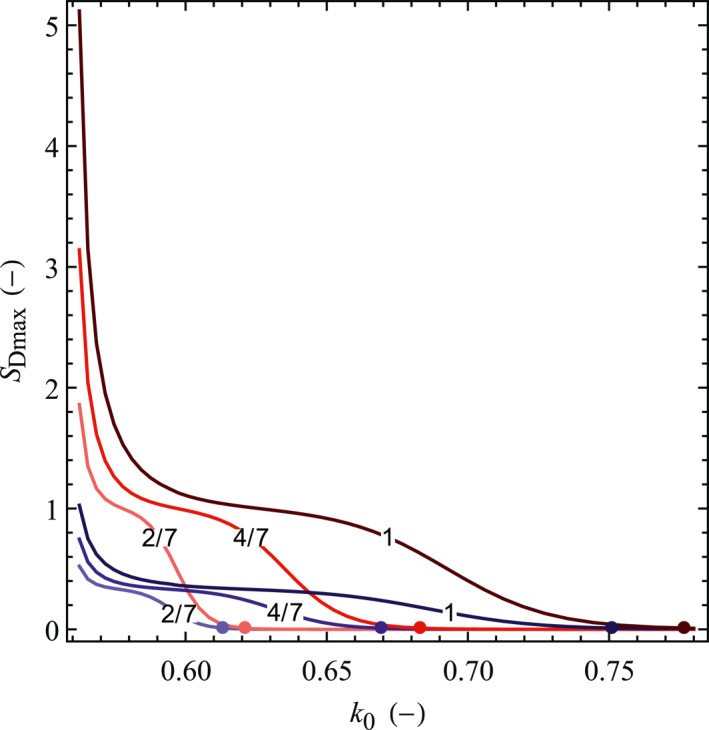
Dimensionless maximum fault slip size (*S*_Dmax_) as a function of the initial stress ratio for several pressure buildups. Dots represent the critical stress ratios. Blue and red color scales for curves and dots correspond to permeable and impermeable faults, respectively, and the number on the curves denotes the dimensionless pressure buildup.

The initial stress state determines the initial *CFS*, which significantly affects the fault stability and fault slip potential. We explore the influence of the initial stress state by applying different values of the horizontal to vertical stress ratio (*k*_0_) while keeping the vertical stress constant (Figure 9). Given that we adopt a value of the static friction coefficient of 0.6, we set a minimum stress ratio of 0.563 to ensure that the initial conditions correspond to *CFS* < 0 (stable fault). We define a critical stress ratio $k_{0}^{c}$ (marked by dots in Figure 9) which corresponds to the threshold of fault slip (*S*_Dmax_ = 0.01), that is, $k_{0}>k_{0}^{c}$ implies a stable fault (negligible rupture size), and $k_{0}<k_{0}^{c}$ a ruptured one for the applied pore pressure change. The dimensionless maximum fault slip size increases with decreasing stress ratio for both the permeable and impermeable faults (Figure 9). In other words, the smaller the stress ratio, the larger the deviatoric stress and thus, the larger the fault slip size. The rate of increase in *S*_Dmax_ with decreasing *k*_0_ is not steady though and is controlled by the cusp‐like shape of *CFS*_D_ (recall Figure 4b). The sharp increase in *S*_Dmax_ once *k*_0_ becomes lower than $k_{0}^{c}$ is due to the progressive failure of the pressurized reservoir. Once the whole reservoir is in failure, the portion of the caprock or bedrock that undergoes failure increases slowly as *k*_0_ decreases (see Figure 4b). As the initial *CFS* approaches 0, the rupture size sharply increases because the asymptotic increase in $\Delta {CFS}_{D}$ within the caprock or bedrock is reached. The maximum rupture size coincides with the minimum in *k*_0_, and for an impermeable fault in the base case scenario (only changing the stress ratio to its minimum) the rupture size is three times greater than the reservoir thickness (*S*_Dmax_ ≈ 3). The rupture size is a lower bound because our model does not incorporate frictional strength weakening (Buijze et al., 2017, 2019; Garagash & Germanovich, 2012), and stress redistribution (De Simone et al., 2017; Sacks et al., 1978) associated with shear slip activation.

**Figure 10:**
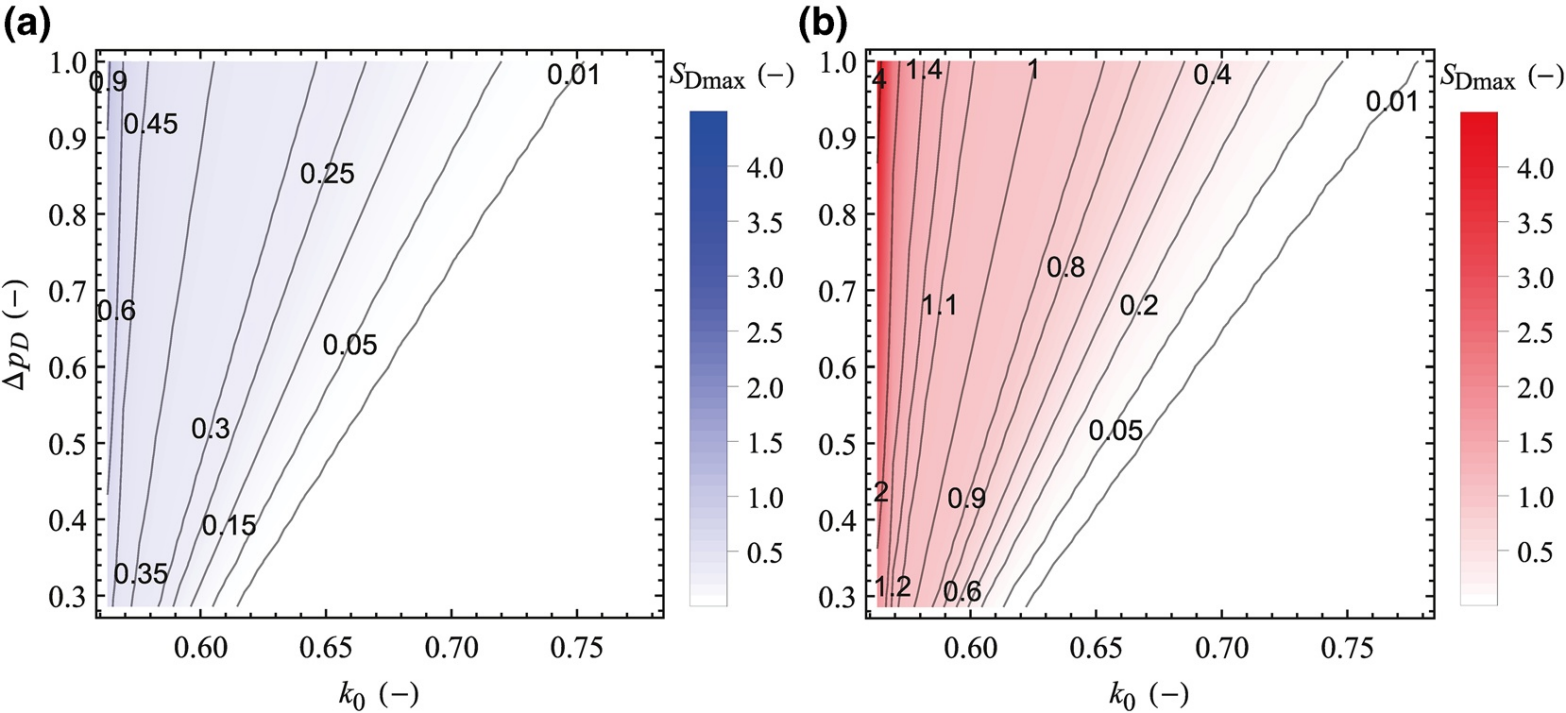
Dimensionless maximum fault slip size (*S*_Dmax_) as a function of the initial stress ratio and dimensionless pressure buildup for (a) permeable and (b) impermeable faults (for comparison purposes, both figures have the same color scale). The numbers on the contours denote the values of *S*_Dmax_.

The effect of operational aspects, expressed as pore pressure changes, is mainly controlled by the injected volume, injection rate, and reservoir boundaries, that is, compartmentalization (Mathias et al., 2009; Nordbotten et al., 2005; Wu et al., 2016, 2018), and it affects the magnitude of induced earthquakes. Therefore, we further explore the (*S*_Dmax_, *k*_0_) space for different values of pressure buildup (Figures 9 and 10). We find that both *S*_Dmax_ and $k_{0}^{c}$ (corresponding to the contour of *S*_Dmax_ = 0.01 in Figure 10) linearly increase with pressure buildup, with the highest increments of *S*_Dmax_ corresponding to the impermeable fault. The contour plots in *S*_Dmax_ with *k*_0_ and ∆*p*_D_ show that the rupture size and the critical stress ratio for an impermeable fault are larger than for a permeable one under any initial and injection conditions (Figure 10). Thus, impermeable faults would rupture at lower initial deviatoric stress and with larger earthquake magnitude. Generally, *S*_Dmax_ for an impermeable fault is 3–5 times greater than for a permeable one under a given *k*_0_ and ∆*p*_D_.

**Figure 11:**
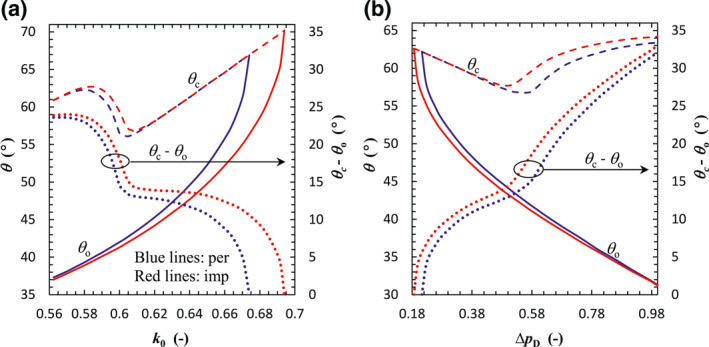
The onset and critical dip angles (*θ*_o_ and *θ*_c_) for fault slip as a function of (a) the initial stress ratio with a dimensionless pressure buildup of 4/7 and (b) the dimensionless pressure buildup with a stress ratio of 0.6. The blue and red lines correspond to the permeable and impermeable faults, respectively. The solid and dashed lines denote *θ*_o_ and *θ*_c_, respectively, while the dotted lines mean the difference between *θ*_c_ and *θ*_o_.

The previous analyses on fault dip show that the onset and critical dip angles (*θ*_o_ and *θ*_c_) are barely related to the fault offset (Figure 8). Nonetheless, Equation 17 suggests that *θ*_o_ and *θ*_c_ depend upon the initial stress ratio *k*_0_ and pressure buildup ∆*p*_D_. Such dependencies are the object of our next analyses. For a given pressure buildup (Table 2), while *θ*_o_ monotonically increases with the stress ratio, *θ*_c_ exhibits a more complex behavior (Figure 11a). *θ*_c_ evolution can be divided into three phases, with an increasing trend as *k*_0_ increases, except for a decreasing branch in the mid‐valued range of *k*_0_. The differential *θ*_c_ – *θ*_o_ decreases with increasing *k*_0_ and equals 0, that is, the onset dip angle coincides with the critical dip angle, at *k*_0_ = 0.674 (i.e., the critical stress ratio in this case) for a permeable fault and at *k*_0_ = 0.694 for an impermeable fault. A direct consequence is that the range of dip angles favorable to slip is reduced for increasing *k*_0_. For *k*_0_ > 0.674 (0.694) the permeable (impermeable) fault is always stable regardless of its inclination (recall Figures 8 and 9). This means that geological sites with a higher in‐situ stress ratio, that is, lower initial deviatoric stress, are intrinsically less prone to fluid injection‐induced seismicity. The onset dip angle is smaller for impermeable faults than for permeable faults. Thus, the range of dip angles favorable to slip is larger for impermeable than for permeable faults, and its difference increases with *k*_0_. The critical stress ratio corresponding to such critical fault dip is also greater for impermeable faults than for permeable ones, similar to the case for an arbitrary fault dip as shown in Figures 9 and 10. The difference in critical stress ratio between Figures 9 and 11 indicates that it increases with dip angle (only for *θ*_o_ < *θ* < *θ*_c_) and peaks at *θ*_c_. Thus, the site characteristics significantly affect the results of the induced seismicity assessment.

In contrast, for a given *k*_0_, the onset dip angle *θ*_o_ monotonically decreases with increasing dimensionless pressure buildup ∆*p*_D_, whereas *θ*_c_ decreases first and then increases with increasing ∆*p*_D_ (Figure 11b). Thus, the differential *θ*_c_ − *θ*_o_ decreases with decreasing ∆*p*_D_ and equals 0 at ∆*p*_D_ = 0.213 (defined as the critical pressure buildup for this case) for a permeable fault and at ∆*p*_D_ = 0.186 for an impermeable fault. Similarly, for ∆*p*_D_ < 0.213 (0.186) the permeable (impermeable) fault is always stable regardless of its inclination. This means that a larger pressure buildup is necessary to induce seismicity, which can be translated into a value of maximum sustainable injection pressure (Bai et al., 2017; Rutqvist et al., 2007; Zhou et al., 2008) to minimize the risk of inducing seismicity. The main difference between permeable and impermeable faults is that the range of dip angles favorable to slip is larger for impermeable than for permeable faults, and the critical ∆*p*_D_ related to *θ*_c_ − *θ*_o_ = 0 is smaller for impermeable than for permeable faults. It implies that an impermeable fault is more likely to induce seismicity in terms of pressure buildup and its maximum sustainable pressure is smaller compared with a permeable fault.

## 5 Discussions

We present an analytical solution to assess fault stability changes ($\Delta {CFS}_{D}$) as a result of reservoir pressurization/depletion. The results are shown in dimensionless form, which generalizes the problem with respect to the pore pressure change. All the stress components, ΔCFS and CFS*,* are normalized by the scaling parameter *C*, which strongly depends on the Poisson's ratio (*ν*). *C* monotonically decreases with the increase of *ν* (Equation 2). Fault stability is obtained by adding $\;\Delta {CFS}_{D}$ to the initial *CFS*_D_, which depends on the initial stress state and pore pressure (Equation 17). The variation in *ν* will change the final *CFS*_D_ and its zero points. An extra calculation shows that the magnitude of *CFS*_D_ and *S*_Dmax_ increases with increasing *ν* for both permeable and impermeable faults. Furthermore, the zero‐offset permeable fault is stabilized when *ν* is lower than 0.24 for the case considered in Table 2, that is, fault dip of 60° and pore pressure buildup of 20 MPa (it is destabilized for *ν* = 0.29 in Figure 4a).

The analytical solution is a useful tool to quickly evaluate the induced seismicity potential of geo‐energy projects. Injection control strategies of the maximum sustainable pressure, which have been validated in CO_2_ sequestration projects (Bai et al., 2017; Rutqvist et al., 2007; Zhou et al., 2008), could benefit from adding this solution in the decision‐making process. A detailed site characterization is needed for its effective application, because the maximum rupture size, and thus, the magnitude of the induced earthquakes, not only depends on operational aspects, like pressure change, but also on the stress state and hydro‐geomechanical characteristics of the fault, such as permeability, strength, offset, and dip. For given initial and injection conditions, the range of dip angles that may undergo failure can be defined, but knowing the hydraulic properties of faults is critical because all the parametric space analyses confirm the unfavorable effect in terms of induced seismicity potential of an impermeable fault*,* and an identical result can be predicted for fluid injection into the right‐hand side of the fault (i.e., the footwall). In particular, low‐permeable faults lead to larger rupture area at lower initial deviatoric stress and at smaller pressure changes (Figures 8, 9, 10, 11). One factor for causing such unfavorable effect is that the differential deformation between the two sides of the impermeable fault distributes in the whole reservoir compartment, that is, the segment P2–P4 for injection into the hanging wall or the segment P1–P3 for injection into the footwall, while the centered segment P2–P3 of a permeable fault always keeps the same deformation.

Pore pressure buildup arising from fluid injection reduces the slip resistance, and thus drives the fault toward failure (Shapiro & Dinske, 2009). The subsequent poroelastic response to adapt to such change tends to balance the system (Figure 3). However, the presence of the corners P1, P2, P3, and P4 (see Figure 1) results in a strong stress concentration (Buijze et al., 2017; Galis et al., 2019), leading to excessive adjustment nearby the corners and fault sliding. Therefore, pore pressure buildup and stress concentration at the corners during reservoir pressurization are the main reasons for fault reactivation and induced seismicity when ignoring thermal effects and geochemical reactions. Stress concentration is mainly controlled by the geometry of corners (Buijze et al., 2017), which changes with fault offset for permeable faults, but is independent of offset for impermeable faults, resulting in different fault stability patterns between permeable and impermeable faults (Figures 5, 6, 7).

Stress concentration not only highlights the effect of the hydraulic nature of faults, but also results in the occurrence of reversed slip at the internal corners P2 and P3 for normal faults with a normal faulting stress regime. In particular, reversed slip becomes the primary slip form for the permeable faults with a large dip angle and offset (Figure 8). In contrast, normal slip will occur and become the primary slip form for a small dip angle and permeable fault in thrust faults with a thrust faulting stress regime, that is, *k*_0_ > 1.

Regarding the criterion for fault slip, one option is to focus on points, that is, when the stress state of one point reaches its failure conditions, for example, CFS > 0, the fault slips. The disadvantage of such a criterion is that the infinite‐induced stress at the corners leads to a misjudgment that even small amounts of injection will induce fault slip (Jansen et al., 2019). Another option consists in considering a minimum unstable patch, like the fault slip size defined in this paper. We have considered a threshold (0.01) for the dimensionless maximum fault slip size, that is, fault slip will not occur unless *S*_Dmax_ > 0.01. Although further research is needed to determine how much the threshold should be, the parametric space analyses and the related conclusions that can be drawn from Section 4 are independent of the threshold value adopted.

Concerning fluid production, the results of $\Delta {CFS}_{D}$ are basically symmetrical to those of injection presented in this paper and are easily obtained by a sign change, because the pressure change is included in the scaling parameter *C*. Thus, the change in fault stability is the opposite to the case of injection and, while fault slip first occurs at the external corners (P1 and P4) during reservoir pressurization, it first happens at the internal corners (P2 and P3) during reservoir depletion for both the permeable and impermeable faults (Jansen et al, 2019). The reverse effect may represent a potential method to control or mitigate induced seismicity: short‐term production followed by an injection phase could improve the stability of the reservoir. Analogously, such an operation could be performed before decommissioning. Moreover, we also observe that the fault slip tendency in the case of production is much smaller than that in injection because $\Delta {CFS}_{D}$ is mainly negative (see Figure 4). Thus, the risk of induced seismicity in the case of injection is higher than that of production in a normal faulting stress regime like the one considered in this study.

Our analytical solution provides an accurate (Figure 2) and fast estimation of the stress variation in the reservoir and its surroundings, which takes into account the increase in stress due to the geological constraints to deformation. The stress variation, which is often called stress arching (Rudnicki 2002; Segall, 1985; Soltanzadeh & Hawkes, 2008), is positive within the reservoir in response to fluid injection. This means that the effective stress reduction is smaller than the pressure buildup (Figure 3). However, many engineering applications in subsurface energy‐related projects neglect the stress arching in assessing fault stability and the effective stress reduction is assumed as equivalent to the pore pressure buildup during injection (e.g., Karvounis et al., 2014). For example, simplified models for CO_2_ sequestration calculate the maximum sustainable pressure as the fracture pressure (Bandilla & Celia, 2017; Mathias et al., 2009; Zhou et al., 2008), neglecting stress arching. We compare the slip tendency analysis estimated by means of our solution with that estimated by neglecting the stress arching in Figure 4. The results show that ignoring the stress arching means overestimating the slip potential for both permeable and impermeable fault in a normal faulting stress regime. This implies an underestimation of the maximum sustainable pressure (i.e., injectivity).

## 6 Conclusion

In recent decades, increasing interest in the subsurface as a source of carbon‐free energy resources has led to an increasing number of induced earthquakes, with some of these earthquakes resulting in the cancellation of projects. To improve the prediction capability of induced seismicity, we have developed an analytical solution to compute the induced stress along both permeable and impermeable faults as a result of reservoir pressurization/depletion. The solution is based on the inclusion theory and has been validated by comparing it with a numerical solution. We have performed a comprehensive analysis on induced seismicity potential due to reservoir pressurization or depletion and obtained the following conclusions:

- The induced seismicity potential of impermeable faults is always larger than that of permeable faults under any initial and injection conditions. Generally, the maximum size of fault undergoing slip for the impermeable faults is 3–5 times greater than that for permeable ones under a given initial stress ratio and pressure buildup. Moreover, an impermeable fault would rupture at a higher stress ratio, that is, less deviatoric stress, and at a smaller pressure buildup than a permeable one.
- Pore pressure buildup and stress concentration at the corners during reservoir pressurization/depletion are the main reasons for fault reactivation and induced seismicity. Stress concentration not only amplifies the effect of the hydraulic properties of faults, but also results in the occurrence of reversed slip at the corners for normal faults with a normal faulting stress regime, and of normal slip for thrust faults with a thrust faulting stress regime.
- The slip potential of permeable faults resulting from reservoir pressurization/depletion increases with the fault offset because of the change in stress concentration, which implies that non‐displaced permeable faults constitute a safer choice for site selection. In contrast, the offset has no impact on the slip potential of impermeable faults, because the effect of stress concentration is always the same.
- For a given pressure buildup, the difference between the critical and onset dip angles, that is, the range of dip angles favorable to slip, reduces for increasing the initial stress ratio and equals to zero at its critical stress ratio. This means that geological sites with a higher in situ stress ratio (lower initial deviatoric stress) are intrinsically less prone to fluid injection‐induced seismicity. This finding is useful for site selection in geo‐energy projects.
- For a given stress ratio, the range of dip angles favorable to slip reduces for decreasing the pressure buildup and equals to zero at its critical pressure buildup as a larger pressure buildup is more likely to induce seismicity. Thus, the methodology of the maximum sustainable injection pressure to minimize the risk of inducing seismicity is feasible and should be available for designing and managing the injection parameters.
- The fault slip potential increases if we ignore the stress arching, that is, assuming the effective stress reduction is equivalent to the pore pressure buildup during injection, for both permeable and impermeable faults in a normal faulting stress regime, which implies that the induced seismicity potential is overestimated and the maximum sustainable pressure is underestimated.
- Our analytical solution includes two limitations resulting from our simplifying assumptions to solve this complex problem: (1) we assume a linear elastic material, which is physically unrealistic for rock materials; in reality, a nonlinear elastic or inelastic deformation cannot be avoided during the reservoir pressurization/depletion; (2) we assume a quasi‐steady‐state pore pressure change in the reservoir and neglect the transient effect of flow, so the calculated induced stress are still overestimated, particularly in the low‐permeable fault zone because pore pressure will eventually diffuse into the portion of the caprock and baserock in contact with the reservoir. Such limitations are worthy to be investigated further and complemented by more detailed numerical solutions

## Supporting information

Supporting Information S1

## Figures and Tables

**Figure B1:**
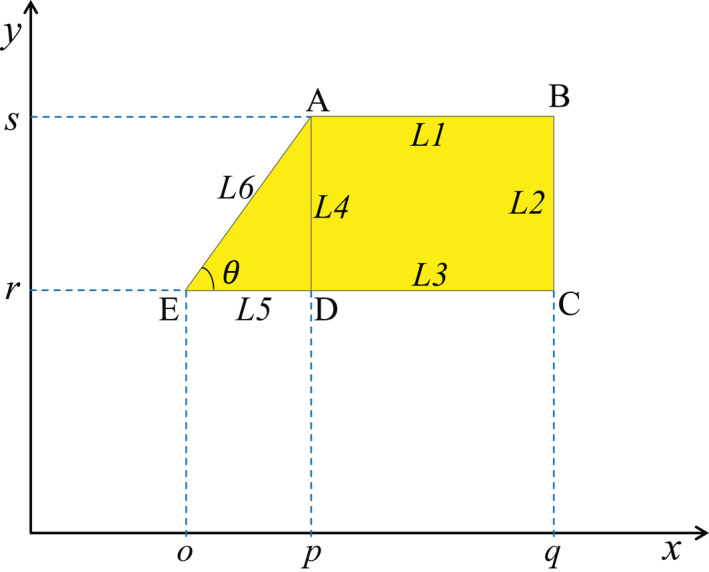
Schematic geometry of triangular and rectangular domains in an arbitrary coordinate system.

## Data Availability

The Python files used to produce most of the figures in this paper and the input files used for the numerical simulations in CODE_BRIGHT are available from the CSIC data repository (https://digital.csic.es/handle/10261/221111).

## Appendix A Inclusion Theory and Induced Stress

### A.1

The fundamental concept of the inclusion theory lies in a series of imaginary steps involving cutting, transforming, and restoring the inclusion itself (Eshelby, 1957; Mura, 1987; Rudnicki, 2011). In the last step, restoring the inclusion to its original shape and size, that is, with zero strain, corresponds to the application of a stress field $\sigma^{*}$ (eigenstress) inside the inclusion, to neutralize the volumetric eigenstrain $\varepsilon^{*}$ that it would undergo if unbounded, and of a simultaneous body force f (restoring force) over the entire matrix, to keep the stress equilibrium (Jansen et al., 2019; Rudnicki, 2011). For the case of uniform pore pressure change inside the inclusion, Δp, then is

(A1)
$$f\left(x,y,z\right)=-\alpha \Delta pn_{\Gamma }\left(x,y,z\right),$$

where the vector f has three components in the coordinate directions for a three‐dimensional (3D) problem, $n_{\Gamma }$ is the unit normal vector pointing outward from the boundary (Γ) of the inclusion, *x*, *y,* and *z* are the Cartesian coordinates, and α is Biot's coefficient. The term −αΔp indicates the normal eigenstress for the 3D scenario.

Affected by the restoring force field, a displacement field $u_{i}\left(x,y,z\right)$ is provoked in the inclusion and its surrounding rock as

(A2)
$$u_{i}\left(x,y,z\right)=-\alpha \Delta p\iint_{\Gamma }{\tilde{g}}_{i}\left(x,y,z,\varsigma ,\xi ,\psi \right)\;\cdot \;n_{\Gamma }\left(\varsigma ,\xi ,\psi \right)\begin{array}{c} d\Gamma \end{array},$$

where

(A3)
$${\tilde{g}}_{i}\left(x,y,z,\varsigma ,\xi ,\psi \right)={\left[{\tilde{g}}_{ix}\left(x,y,z,\varsigma ,\xi ,\psi \right),{\tilde{g}}_{iy}\left(x,y,z,\varsigma ,\xi ,\psi \right),{\tilde{g}}_{iz}\left(x,y,z,\varsigma ,\xi ,\psi \right)\right]}^{T},$$

and ${\tilde{g}}_{ij}\left(x,y,z,\varsigma ,\xi ,\psi \right)$ is the Green's function describing the displacement at any point (x,y,z) under a unit body force at point (ς,ξ,ψ), ς,ξ,ψ are the coordinate values on Γ, and subscripts *i* and *j* are free indexes with i,j∈(x,y,z) in the 3D Cartesian space.

For an infinite elastic unbounded domain, ${\tilde{g}}_{ij}\left(x,y,z,\varsigma ,\xi ,\psi \right)$ can be expressed as (Love, 1944; Mura, 1987)

(A4)
$${\tilde{g}}_{ij}\left(x,y,z,\varsigma ,\xi ,\psi \right)=\frac{\delta_{ij}}{4\pi \mu \tilde{R}}-\frac{1}{16\pi \mu \left(1-\nu \right)}\frac{\partial^{2}}{\partial x_{i}\partial x_{j}}\tilde{R},$$

where μ and ν are the shear modulus and Poisson's ratio, respectively, $\delta_{ij}$ is the Kronecker delta, which equals 1 if *i* = *j* or 0 if *i* ≠ *j*, and

(A5)
$${\tilde{R}}^{2}={\left(x-\varsigma \right)}^{2}+{\left(y-\xi \right)}^{2}+{\left(z-\psi \right)}^{2}.$$

Equation A2 is a standard surface integral and can be transformed into a volume integral by applying the Gauss's divergence theorem

(A6)
$$u_{i}\left(x,y,z\right)=-\alpha \Delta p{∭}_{\Omega }\nabla \cdot {\tilde{g}}_{i}\left(x,y,z,\varsigma ,\xi ,\psi \right)\begin{array}{c} d\Omega \end{array},$$

where Ω means the inclusion volume. Note that now ς,ξ,ψ in Equation A6 denote the coordinate values in the domain Ω.

Under the assumption of plane strain and integrating Equation A4 along the out‐of‐plane dimension (*z*) yields (Jansen et al., 2019; Mura, 1987)

(A7)
$${\tilde{g}}_{ij}\left(x,y,\varsigma ,\xi \right)=\frac{1}{8\pi \mu \left(1-\nu \right)}\left[\frac{{\bar{x}}_{i}\cdot {\bar{x}}_{j}}{R^{2}}-\left(3-4\nu \right)\delta_{ij}\;\ln\;R\right]\;i,j\in \left(x,y\right),$$

where

(A8)
$${\bar{x}}_{i}=\left|x-\varsigma \right|\begin{array}{c} \text{or}\begin{array}{c} \left|y-\xi \right| \end{array} \end{array},$$

(A9)
$$R^{2}={\left(x-\varsigma \right)}^{2}+{\left(y-\xi \right)}^{2}.$$

Equation A6 can be simplified into

(A10)
$$u_{i}\left(x,y\right)=-\alpha \Delta p\iint_{\Omega }\frac{\partial {\tilde{g}}_{ix}}{\partial x}+\frac{\partial {\tilde{g}}_{iy}}{\partial y}d\Omega =D\iint_{\Omega }g_{i}\left(x,y,\varsigma ,\xi \right)d\Omega ,$$

where $g_{i}\left(x,y,\varsigma ,\xi \right)$ is

(A11)
$$g_{x}\left(x,y,\varsigma ,\xi \right)=\frac{x-\varsigma }{2R^{2}},$$

(A12)
$$g_{y}\left(x,y,\varsigma ,\xi \right)=\frac{y-\xi }{2R^{2}},$$

and *D* is a dimensionless scaling parameter

(A13)
$$D=\frac{\left(1-2\nu \right)\alpha \Delta p}{2\pi \left(1-\nu \right)\mu }=\frac{\left(1+\nu \right)\alpha \Delta p}{3\pi \left(1-\nu \right)K},$$

where *K* is the bulk modulus.

For an isotropic linear elastic material, the Hooke's equation relating the stress *σ*_*ij*_ to the strain *ε*_*ij*_ tensor is

(A14)
$$\sigma_{ij}=2\mu \varepsilon_{ij}+\frac{2\mu \nu }{1-2\nu }\delta_{ij}\varepsilon_{kk},$$

where $\epsilon_{kk}$ is the volumetric strain and *ε*_*ij*_ equals the symmetric part of the displacement gradient

(A15)
$$\varepsilon_{ij}=\frac{1}{2}\left(\frac{\partial u_{i}}{\partial x_{j}}+\frac{\partial u_{j}}{\partial x_{i}}\right).$$

Substituting Equations A10 into A15, and taking the results into Equation A14, yields the expression of the stress field in the whole matrix induced by the restoring force **f**

(A16)
$$\sigma_{ij}\left(x,y\right)=C\iint_{\Omega }g_{ij}\left(x,y,\varsigma ,\xi \right)d\Omega ,$$

where C=μD is a scaling parameter and $g_{ij}\left(x,y,\varsigma ,\xi \right)$ is the Green's function for stress at (*x*, *y*) given a unit point force at (ς,ξ)

(A17)
$$g_{xx}\left(x,y,\varsigma ,\xi \right)=2\frac{\partial g_{x}}{\partial x}=\frac{{\left(y-\xi \right)}^{2}-{\left(x-\varsigma \right)}^{2}}{R^{4}},$$

(A18)
$$g_{yy}\left(x,y,\varsigma ,\xi \right)=2\frac{\partial g_{y}}{\partial y}=\frac{{\left(x-\varsigma \right)}^{2}-{\left(y-\xi \right)}^{2}}{R^{4}},$$

(A19)
$$g_{xy}\left(x,y,\varsigma ,\xi \right)=2\frac{\partial g_{x}}{\partial y}=-2\frac{\left(x-\varsigma \right)\left(y-\xi \right)}{R^{4}}.$$

For the inclusion, the stress field, however, is also affected by the eigenstress resulting from the application of the surface traction to bring the inclusion back to its initial configuration (Eshelby, 1957). Since the surface traction depends on the geometry of the inclusion, such eigenstress is different from that of the 3D scenario for the case of the plane strain problem. Regarding that the eigenstrain is a pure dilatational strain, only the normal components of eigenstress have a finite value (Eshelby, 1957; Soltanzadeh & Hawkes, 2008), thus

(A20)
$$\sigma_{ij}\left(x,y\right)=C\iint_{\Omega }g_{ij}\left(x,y,\varsigma ,\xi \right)d\Omega +\sigma^{*}\delta_{ij}\delta_{\Omega }=CG_{ij}x,y+\sigma^{*}\delta_{ij}\delta_{\Omega },$$

where $G_{ij}\left(x,y\right)$ denotes the surface integral of Green's function $g_{ij}\left(x,y,\varsigma ,\xi \right)$, and $\delta_{\Omega }$ is the modified Kronecker delta

(A21)
$$\delta_{\Omega }=\left\{\begin{array}{cc} 1 & \;\text{if}\;\left(x,y\right)\;\in \;\Omega ,\\ 0 & \;\text{if}\;(x,y)\;\notin \;\Omega . \end{array}\right)$$

From the perspective of stress arching effect, the induced stress field caused by pore pressure change in the inclusion can be described as (Soltanzadeh & Hawkes, 2008)

(A22)
$$\sigma_{ij}=-\gamma_{ij}\alpha \Delta p,$$

where $\gamma_{ij}$ is the normalized stress arching ratio, which depends on the geometry of the inclusion. The minus means that injection corresponds to compression.

For the ellipsoidal inclusions, the stress and strain fields are uniform for all points inside the inclusion (Eshelby, 1957; Rudnicki, 1999). And for the case in which the inclusion extends infinite in one direction (implying that we can apply the plane strain assumption), such as the elliptic cylindrical inclusion, the sum of the stress arching ratio in the other two directions is a constant (Soltanzadeh, 2009; Soltanzadeh & Hawkes, 2008)

(A23)
$$\gamma_{xx}+\gamma_{yy}=\frac{\left(1-2\nu \right)}{1-\nu }.$$

According to Equations A20–A23, we find that

(A24)
$$C\left[G_{xx}\left(x,y\right)+G_{yy}\left(x,y\right)\right]+2\sigma^{*}=-\frac{1-2\nu }{1-\nu }\alpha \Delta p.$$

Given that $g_{xx}\left(x,y,\varsigma ,\xi \right)=-g_{yy}\left(x,y,\varsigma ,\xi \right)$ (Equations A17 and A18), thus

(A25)
$$\sigma^{*}=-\frac{1-2\nu }{2\left(1-\nu \right)}\alpha \Delta p.$$

Although Equation A25 is derived for an elliptic cylindrical inclusion, it is valid for all the geometric inclusions that can be regarded as plane strain problem, because the inclusion only undergoes a pure dilatational deformation. Introducing Equation A25 into Equation A20, we obtain the final expression of the induced stress tensor both in the inclusion and its surrounding rock, as given in Equation 1.

## Appendix B Surface Integral of Green's Function for Stress

### B.1

In this appendix, we solve the surface integrals of Equation 1. The entire inclusion is divided into two trapezoids by the fault. The surface integral of one function over a trapezoid can be regarded as the sum of the integrations over a triangle and a rectangle (Figure B1). The integrand function is the Green's function for stress given in Equations (A17), (A18), (A19). We need to consider the existence of singularities for values of *ς* and *ξ* equal *x* and *y*, respectively, where the Green's function becomes infinite. This only occurs for points (*x*, *y*) located inside the inclusion, for which the integral becomes improper. Thus, we perform the regular bounded integral for (*x*, *y*) located outside the inclusion, while for (*x*, *y*) located inside the inclusion we solve the improper integral by excluding a neighborhood of the singularity. To generalize the integration of Green's function, we apply an arbitrary coordinate system, as shown in Figure B1. After solving the surface integrals over the triangular and rectangular domains, the solutions are transformed into the coordinate system of Figure 1.

### Rectangular Inclusion

For (*x*, *y*) located outside of the rectangle, we calculate the surface integral of the Green's function for horizontal stress $g_{xx}\left(x,y,\varsigma ,\xi \right)$ using standard techniques and we obtain

(B1)
$$\begin{array}{ccc} G_{xx}^{\text{out}}\left(x,y\right) & = & \int_{r}^{s}\int_{p}^{q}g_{xx}\left(x,y,\varsigma ,\xi \right)d\varsigma d\xi \\ & = & \int_{r}^{s}-{\left[\frac{x-\varsigma }{{\left(x-\varsigma \right)}^{2}+{\left(y-\xi \right)}^{2}}\right]}_{p}^{q}d\xi \\ & = & {\left[a\tan\frac{y-\xi }{x-q}-a\tan\frac{y-\xi }{x-p}\right]}_{r}^{s}\\ & = & a\tan\frac{y-s}{x-q}-a\tan\frac{y-s}{x-p}-a\tan\frac{y-r}{x-q}+a\tan\frac{y-r}{x-p}. \end{array}$$

If (*x*, *y*) is located in the rectangle, we need to consider the improper integration of Green's function. Note that if the standard integration (like in equation (B1)) is applied for such points, the result depends on the order of integration, because Fubini's theorem (DiBenedetto, 2016) does not hold in this case. We solve the improper integral excluding a neighborhood of the singular point (*ς, ξ*) = (*x, y*), which makes the integrand function being bounded. We observe that $g_{xx}\left(x,y,\varsigma ,\xi \right)$ is antisymmetric with respect to the line *y*−*ξ* = *x*–*ς*, that is, $g_{xx}\left(x,y,\varsigma ,\xi \right)=-g_{xx}\left(y,x,\xi ,\varsigma \right)$. This means that the integral is zero for a square domain centered in the singular point, because the contribution from the triangle above the symmetry line cancels with the contribution from the triangle below. Therefore, we can exclude a square neighborhood of any size contained in the domain Ω and centered in the singular point. In the rest of the domain, Fubini's theorem holds and we can apply the standard sequential integration (Equation B1). For the square domain, the actual contribution is zero while standard integration technique gives π, which can be checked by substituting *y*−*r* = *x*−*p* = *s*−*y* = *q*−*x* into Equation (B1). Therefore, we must remove the offending contribution, so that the integral for points inside the inclusion is

(B2)
$$G_{xx}^{\text{in}}\left(x,y\right)=G_{xx}^{\text{out}}\left(x,y\right)-\pi .$$

Note that this result is independent on the order of integration.

Equations B1 and B2 only differ for the last term −π, which is a result of the improper integral for points located inside the reservoir. We therefore express the surface integral in the general form

(B3)
$$G_{xx}\left(x,y\right)=a\tan\frac{y-r}{x-p}-a\tan\frac{y-r}{x-q}-a\tan\frac{y-s}{x-p}+a\tan\frac{y-s}{x-q}-\pi \delta_{\Omega },$$

with *δ*_Ω_ defined in Equation A21.

There are four singularities at the corners (A, B, C, and D) of the rectangular inclusion domain (Figure B1), where the arguments in Equation B3 become indefinite (i.e., 0/0). The integral is in fact not defined at these points, and it is discontinuous there (different values are obtained when approaching it from one side or the other).

The same procedure can be applied to integrate the Green's function for vertical stress $g_{yy}\left(x,y,\varsigma ,\xi \right)$. Since $g_{yy}\left(x,y,\varsigma ,\xi \right)=-g_{xx}\left(x,y,\varsigma ,\xi \right)$ (see Equations A17 and A18), it follows that

(B4)
$$G_{yy}\left(x,y\right)=\int_{r}^{s}\int_{p}^{q}g_{yy}\left(x,y,\varsigma ,\xi \right)\begin{array}{c} d\varsigma d\xi \end{array}=-G_{xx}\left(x,y\right).$$

To integrate the Green's function for shear stress $g_{xy}\left(x,y,\varsigma ,\xi \right)$, we follow the same procedure used to integrate $g_{xx}\left(x,y,\varsigma ,\xi \right)$. We observe that $g_{xy}\left(x,y,\varsigma ,\xi \right)$ is antisymmetric with respect to *y*−*ξ* = 0 or *x*−*ς* = 0, therefore the integral over a square domain centered in the singular point is 0. We also observe that the standard sequential integration technique in this case gives

(B5)
$$\begin{array}{ccc} G_{xy}\left(x,y\right) & = & \int_{p}^{q}\int_{r}^{s}g_{xy}\left(x,y,\varsigma ,\xi \right)d\xi d\varsigma \\ & = & \int_{p}^{q}{\left[\frac{-\left(x-\varsigma \right)}{{\left(x-\varsigma \right)}^{2}+{\left(y-\xi \right)}^{2}}\right]}_{r}^{s}d\varsigma =\frac{1}{2}{\left[\ln\frac{{\left(x-\varsigma \right)}^{2}+{\left(y-s\right)}^{2}}{{\left(x-\varsigma \right)}^{2}+{\left(y-r\right)}^{2}}\right]}_{p}^{q}\\ & = & \frac{1}{2}\ln\frac{\left[{\left(x-q\right)}^{2}+{\left(y-s\right)}^{2}\right]\left[{\left(x-p\right)}^{2}+{\left(y-r\right)}^{2}\right]}{\left[{\left(x-q\right)}^{2}+{\left(y-r\right)}^{2}\right]\left[{\left(x-p\right)}^{2}+{\left(y-s\right)}^{2}\right]}. \end{array}$$

Application of Equation B5 for a square neighborhood of the singular point gives 0, which demonstrates that Equation B5 is valid for both the cases of (*x*, *y*) located outside or inside the inclusion.

In Equation B5, there are four singularities at the corners A, B, C, and D.

### Triangular Inclusion

For the integration over a triangular inclusion, we apply the same technique adopted for the case of the rectangular inclusion. As an example, the integral of the Green's function for horizontal stress is

(B6)
$$\begin{array}{ccc} G_{xx}\left(x,y\right) & = & \int_{r}^{s}\int_{\xi \;\cot\;\theta }^{p}\frac{{\left(y-\xi \right)}^{2}-{\left(x-\varsigma \right)}^{2}}{{\left[{\left(x-\varsigma \right)}^{2}+{\left(y-\xi \right)}^{2}\right]}^{2}}d\varsigma d\xi -\pi \delta_{\Omega }\\ & = & \int_{r}^{s}-{\left[\frac{x-\varsigma }{{\left(x-\varsigma \right)}^{2}+{\left(y-\xi \right)}^{2}}\right]}_{\xi \;\cot\;\theta }^{p}d\xi -\pi \delta_{\Omega }\\ & = & {\left[a\tan\frac{y-\xi }{x-p}\right]}_{r}^{s}+\int_{r}^{s}\frac{x-\xi \;\cot\;\theta }{{\left(x-\xi \;\cot\;\theta \right)}^{2}+{\left(y-\xi \right)}^{2}}d\xi -\pi \delta_{\Omega }, \end{array}$$

where the last term $-\pi \delta_{\Omega }$ takes into account the effect of the improper integral for points located inside the inclusion. The integration of the second term on the right‐hand side of Equation B6 is nontrivial and a potential solution can be obtained by the following transformation:

(B7)
$$\begin{array}{ccc} x-\xi \;\cot\;\theta & = & \left[\left(x-\xi \;\cot\;\theta \right){\cos\;}^{2}\;\theta +\left(y-\xi \right)\sin\;\theta \;\cos\;\theta \right]\\ & & +\left[\left(x-\xi \;\cot\;\theta \right){\sin\;}^{2}\;\theta -\left(y-\xi \right)\sin\;\theta \;\cos\;\theta \right]. \end{array}$$

Substituting Equation B7 into Equation B6 and after some derivations, we obtain

(B8)
$$\begin{array}{ccc} \int_{r}^{s}\frac{x-\xi \;\cot\;\theta }{{\left(x-\xi \;\cot\;\theta \right)}^{2}+{\left(y-\xi \right)}^{2}}d\xi & = & -\frac{\sin\;\theta \;\cos\;\theta }{2}{\left[\ln\left[{\left(x-\xi \;\cot\;\theta \right)}^{2}+{\left(y-\xi \right)}^{2}\right]\right]}_{r}^{s}\\ & & -{\sin\;}^{2}\;\theta {\left[a\tan\frac{\left(x-\xi \;\cot\;\theta \right)\cot\;\theta +\left(y-\xi \right)}{x-y\;\cot\;\theta }\right]}_{r}^{s}. \end{array}$$

The final expression of the integration of the Green's function for horizontal stress over a triangular inclusion domain is

(B9)
$$\begin{array}{ccc} G_{xx}\left(x,y\right) & = & a\tan\frac{y-s}{x-p}-a\tan\frac{y-r}{x-p}-\frac{\sin\;\theta \;\cos\;\theta }{2}\ln\frac{f_{2}\left(x,y,s\right)}{f_{2}\left(x,y,r\right)}\\ & & -\left[f_{1}\left(x,y,s\right)-f_{1}\left(x,y,r\right)\right]{\sin\;}^{2}\;\theta -\pi \delta_{\Omega }, \end{array}$$

where functions *f*_1_ and *f*_2_ are

(B10)
$$f_{1}\left(x,y,\hat{y}\right)=a\tan\frac{\left(x-\hat{y}\cot\;\theta \right)\cot\;\theta +\left(y-\hat{y}\right)}{x-y\;\cot\;\theta },$$

(B11)
$$f_{2}\left(x,y,\hat{y}\right)={\left(x-\hat{y}\cot\;\theta \right)}^{2}+{\left(y-\hat{y}\right)}^{2}.$$

Similarly, integrations of the Green's function for the vertical and shear stress components over a triangular inclusion domain are

(B12)
$$G_{yy}\left(x,y\right)=\int_{r}^{s}\begin{array}{c} \int_{\xi \;\cot\;\theta }^{p}g_{yy}\left(x,y,\varsigma ,\xi \right)\begin{array}{c} d\varsigma d\xi \end{array} \end{array}=-G_{xx}\left(x,y\right),$$

(B13)
$$\begin{array}{ccc} G_{xy}\left(x,y\right) & = & \int_{r}^{s}\int_{\xi \;\cot\;\theta }^{p}g_{xy}\left(x,y,\varsigma ,\xi \right)d\varsigma d\xi \\ & = & \left[f_{1}\left(x,y,s\right)-f_{1}\left(x,y,r\right)\right]\sin\;\theta \;\cos\;\theta \\ & & -\frac{{\sin\;}^{2}\;\theta }{2}\ln\frac{f_{2}\left(x,y,s\right)}{f_{2}\left(x,y,r\right)}+\frac{1}{2}\ln\frac{f_{3}\left(x-p,y-s\right)}{f_{3}\left(x-p,y-r\right)}, \end{array}$$

where function *f*_3_ is

(B14)
$$f_{3}\left(x-\hat{x},y-\hat{y}\right)={\left(x-\hat{x}\right)}^{2}+{\left(y-\hat{y}\right)}^{2},$$

The singularities are located at the three corners A, D, and E in this case.

### Trapezoidal Inclusion

We apply the superposition principle and obtain the integrations of the Green's function for the stress components over a trapezoid as a combination of the above integrals for rectangular and triangular inclusions as

(B15)
$$\begin{array}{ccc} G_{xx}\left(x,y\right) & = & \int_{r}^{s}\int_{\xi \;\cot\;\theta }^{p}g_{xx}\left(x,y,\varsigma ,\xi \right)d\varsigma d\xi +\int_{r}^{s}\int_{p}^{q}g_{xx}\left(x,y,\varsigma ,\xi \right)d\varsigma d\xi \\ & = & a\tan\frac{y-s}{x-q}-a\tan\frac{y-r}{x-q}-\frac{\sin\;\theta \;\cos\;\theta }{2}\ln\frac{f_{2}\left(x,y,s\right)}{f_{2}\left(x,y,r\right)}\\ & & -\left[f_{1}\left(x,y,s\right)-f_{1}\left(x,y,r\right)\right]{\sin\;}^{2}\;\theta -\pi \delta_{\Omega }, \end{array}$$

(B16)
$$G_{yy}\left(x,y\right)=\int_{r}^{s}\begin{array}{c} \int_{\xi \;\cot\;\theta }^{p}g_{yy}\left(x,y,\varsigma ,\xi \right)\begin{array}{c} d\varsigma d\xi \end{array} \end{array}+\int_{r}^{s}\int_{p}^{q}g_{yy}\left(x,y,\varsigma ,\xi \right)\begin{array}{c} d\varsigma d\xi \end{array}=-G_{xx}\left(x,y\right),$$

(B17)
$$\begin{array}{ccc} G_{xy}\left(x,y\right) & = & \int_{r}^{s}\int_{\xi \;\cot\;\theta }^{p}g_{xy}\left(x,y,\varsigma ,\xi \right)d\varsigma d\xi +\int_{r}^{s}\int_{p}^{q}g_{xx}\left(x,y,\varsigma ,\xi \right)d\varsigma d\xi \\ & = & \left[f_{1}\left(x,y,s\right)-f_{1}\left(x,y,r\right)\right]\sin\;\theta \;\cos\;\theta \\ & & -\frac{{\sin\;}^{2}\;\theta }{2}\ln\frac{f_{2}\left(x,y,s\right)}{f_{2}\left(x,y,r\right)}+\frac{1}{2}\ln\frac{f_{3}\left(x-q,y-s\right)}{f_{3}\left(x-q,y-r\right)}. \end{array}$$

Note that the last term in Equation B15 is a consequence of having improper integrals either for the rectangle or the triangle, i.e., (*x*, *y*) falls within the rectangle or the triangle. In Equations B15–B17, the singularities are located at the four corners A, B, C, and E of the trapezoid.

### Application into a Specific Coordinate System

For a permeable fault, pore pressure changes at both sides of the fault during fluid injection or production and thus, the inclusion is composed of two trapezoids. The analytical expressions of integration of the Green's function for the stress components in the general coordinate system is transformed into the coordinate system of Figure 1 as

(B18)
$$\begin{array}{ccc} G_{xx}\left(x,y\right) & = & \int_{a}^{-b}\int_{\xi \;\cot\;\theta }^{-b\;\cot\;\theta }g_{xx}\left(x,y,\varsigma ,\xi \right)d\varsigma d\xi +\int_{a}^{-b}\int_{-b\;\cot\;\theta }^{-c}g_{xx}\left(x,y,\varsigma ,\xi \right)d\varsigma d\xi \\ & & +\int_{-a}^{b}\int_{\xi \;\cot\;\theta }^{b\;\cot\;\theta }g_{xx}\left(x,y,\varsigma ,\xi \right)d\varsigma d\xi +\int_{-a}^{b}\int_{b\;\cot\;\theta }^{d}g_{xx}\left(x,y,\varsigma ,\xi \right)d\varsigma d\xi \\ & = & \int_{a}^{-b}\int_{\xi \;\cot\;\theta }^{-c}g_{xx}\left(x,y,\varsigma ,\xi \right)d\varsigma d\xi +\int_{-a}^{b}\int_{\xi \;\cot\;\theta }^{d}g_{xx}\left(x,y,\varsigma ,\xi \right)d\varsigma d\xi \\ & = & a\tan\frac{y+b}{x+c}-a\tan\frac{y-a}{x+c}+a\tan\frac{y-b}{x-d}-a\tan\frac{y+a}{x-d}\\ & & -\left[f_{1}\left(x,y,-b\right)-f_{1}\left(x,y,a\right)+f_{1}\left(x,y,b\right)-f_{1}\left(x,y,-a\right)\right]{\sin\;}^{2}\;\theta \\ & & -\frac{\sin\;\theta \;\cos\;\theta }{2}\ln\frac{f_{2}\left(x,y,-b\right)f_{2}\left(x,y,b\right)}{f_{2}\left(x,y,a\right)f_{2}\left(x,y,-a\right)}-\pi \delta_{\Omega }, \end{array}$$

(B19)
$$G_{yy}\left(x,y\right)=\int_{a}^{-b}\int_{\xi \;\cot\;\theta }^{-c}g_{yy}\left(x,y,\varsigma ,\xi \right)\begin{array}{c} d\varsigma d\xi \end{array}+\int_{-a}^{b}\int_{\xi \;\cot\;\theta }^{d}g_{yy}\left(x,y,\varsigma ,\xi \right)\begin{array}{c} d\varsigma d\xi \end{array}=-G_{xx}\left(x,y\right),$$

(B20)
$$\begin{array}{ccc} G_{xy}\left(x,y\right) & = & \int_{a}^{-b}\int_{\xi \;\cot\;\theta }^{-c}g_{xy}\left(x,y,\varsigma ,\xi \right)d\varsigma d\xi +\int_{-a}^{b}\int_{\xi \;\cot\;\theta }^{d}g_{xy}\left(x,y,\varsigma ,\xi \right)d\varsigma d\xi \\ & = & \left[f_{1}\left(x,y,-b\right)-f_{1}\left(x,y,a\right)+f_{1}\left(x,y,b\right)-f_{1}\left(x,y,-a\right)\right]\sin\;\theta \;\cos\;\theta \\ & & -\frac{{\sin\;}^{2}\;\theta }{2}\ln\frac{f_{2}\left(x,y,-b\right)f_{2}\left(x,y,b\right)}{f_{2}\left(x,y,a\right)f_{2}\left(x,y,-a\right)}+\frac{1}{2}\ln\frac{f_{3}\left(x+c,y+b\right)f_{3}\left(x-d,y-b\right)}{f_{3}\left(x+c,y-a\right)f_{3}\left(x-d,y+a\right)}. \end{array}$$

If *c* and *d* have finite value, the inclusion represents a finite reservoir and there are eight singularities for the solution: the four corners P1, P2, P3, and P4 located on the fault (Figure 1) and the four corners on the outer boundary of the faulted reservoir. If *c* = *d* = ∞, the reservoir is infinite and there are only the four singularities located on the fault.

For a vertical fault (θ=90), such integral solutions read as

(B21)
$$\begin{array}{ccc} G_{xx}\left(x,y\right) & = & -G_{yy}\left(x,y\right)\\ & = & \int_{-b}^{a}\int_{-c}^{0}g_{xx}\left(x,y,\varsigma ,\xi \right)d\varsigma d\xi +\int_{-a}^{b}\int_{0}^{d}g_{xx}\left(x,y,\varsigma ,\xi \right)d\varsigma d\xi \\ & = & a\tan\frac{y-a}{x}-a\tan\frac{y-a}{x+c}-a\tan\frac{y+b}{x}+a\tan\frac{y+b}{x+c}\\ & & +a\tan\frac{y-b}{x-d}-a\tan\frac{y-b}{x}-a\tan\frac{y+a}{x-d}+a\tan\frac{y+a}{x}-\pi \delta_{\Omega }, \end{array}$$

(B22)
$$\begin{array}{ccc} G_{xy}\left(x,y\right) & = & \int_{-c}^{0}\int_{-b}^{a}g_{xy}\left(x,y,\varsigma ,\xi \right)d\xi d\varsigma +\int_{0}^{d}\int_{-a}^{b}g_{xy}\left(x,y,\varsigma ,\xi \right)d\xi d\varsigma \\ & = & \frac{1}{2}\ln\frac{f_{3}\left(x,y-a\right)f_{3}\left(x+c,y+b\right)f_{3}\left(x-d,y-b\right)f_{3}\left(x,y+a\right)}{f_{3}\left(x,y+b\right)f_{3}\left(x+c,y-a\right)f_{3}\left(x-d,y+a\right)f_{3}\left(x,y-b\right)}, \end{array}$$

and for a zero offset fault in which *a* = *b*, we have

(B23)
$$\begin{array}{c} G_{xx}\left(x,y\right)=-G_{yy}\left(x,y\right)\\ =a\tan\frac{y+b}{x+c}-a\tan\frac{y-a}{x+c}+a\tan\frac{y-b}{x-d}-a\tan\frac{y+a}{x-d}-\pi \delta_{\Omega }, \end{array}$$

(B24)
$$G_{xy}\left(x,y\right)=\frac{1}{2}\ln\frac{f_{3}\left(x+c,y+b\right)f_{3}\left(x-d,y-b\right)}{f_{3}\left(x+c,y-a\right)f_{3}\left(x-d,y+a\right)}.$$

## Appendix C Induced Stress on the Fault Plane

### C.1

The expression for normal and tangential stress on the fault plane can be found by transforming the main coordinate system into a coordinate system placed on the fault and oriented along it. We first operate the translation, thus the horizontal and vertical integral solutions of the Green's function for the stress components on the fault plane are evaluated by setting x=ycotθ in Equations B18–B20, such that

(C1)
$$\begin{array}{c} G_{xx}\left(y\;\cot\;\theta ,y\right)=-G_{yy}\left(y\;\cot\;\theta ,y\right)\\ =-\frac{\pi }{2}\left[\mathrm{sgn}\left(y+b\right)-\mathrm{sgn}\left(y-a\right)+\mathrm{sgn}\left(y-b\right)-\mathrm{sgn}\left(y+a\right)\right]{\sin\;}^{2}\;\theta \\ +a\tan\frac{y+b}{y\;\cot\;\theta +c}-a\tan\frac{y-a}{y\;\cot\;\theta +c}+a\tan\frac{y-b}{y\;\cot\;\theta -d}\\ -a\tan\frac{y+a}{y\;\cot\;\theta -d}-\frac{\sin\;\theta \;\cos\;\theta }{2}\ln\frac{f_{4}\left(y,b\right)}{f_{4}\left(y,a\right)}-\pi \delta_{\Omega }, \end{array}$$

(C2)
$$\begin{array}{ccc} G_{xy}\left(y\;\cot\;\theta ,y\right) & = & \frac{\pi }{2}\left[\mathrm{sgn}\left(y+b\right)-\mathrm{sgn}\left(y-a\right)+\mathrm{sgn}\left(y-b\right)-\mathrm{sgn}\left(y+a\right)\right]\sin\;\theta \;\cos\;\theta \\ & & +\frac{1}{2}\ln\frac{f_{3}\left(y\;\cot\;\theta +c,y+b\right)f_{3}\left(y\;\cot\;\theta -d,y-b\right)}{f_{3}\left(y\;\cot\;\theta +c,y-a\right)f_{3}\left(y\;\cot\;\theta -d,y+a\right)}-\frac{{\sin\;}^{2}\;\theta }{2}\ln\frac{f_{4}\left(y,b\right)}{f_{4}\left(y,a\right)}. \end{array}$$

where sgn (•) is the sign function defined as 1 if (*•*) > 0, 0 if (*•*) = 0 or −1 if (*•*) <0, and function *f*_4_ is

(C3)
$$f_{4}\left(y,\hat{y}\right)={\left(y+\hat{y}\right)}^{2}{\left(y-\hat{y}\right)}^{2},$$

Substituting Equations C1 and C2 into Equation 1 yields the *x–y* planar induced stress along the fault plane. Subsequently, applying the stress transformation with axis rotation to transform the coordinate system, one can obtain the expression of induced normal $\sigma_{n}\left(y\cot\theta ,\;y\right)\;$ and tangential τ(ycotθ,y) stress components along a fault plane with an arbitrary dip angle. Considering the sign convention and geometry adopted here, such stress transformation equations are

(C4)
$$\sigma_{n}\left(y\;\cot\;\theta ,y\right)=\frac{\sigma_{xx}+\sigma_{yy}}{2}+\frac{\sigma_{xx}-\sigma_{yy}}{2}\cos\left(2\theta +\pi \right)+\sigma_{xy}\;\sin\left(2\theta +\pi \right),$$

(C5)
$$\tau \left(y\;\cot\;\theta ,y\right)=-\frac{\sigma_{xx}-\sigma_{yy}}{2}\sin\left(2\theta +\pi \right)+\sigma_{xy}\;\cos\left(2\theta +\pi \right).$$

Substituting Equations C1, C2, and 1 into Equations C4 and C5 and normalizing by the scaling parameter *C* (Equation 2), the dimensionless induced normal ${\bar{\sigma }}_{n}\left(y\cot\theta ,\;y\right)$ and tangential $\bar{\tau }\left(y\cot\theta ,\;y\right)$ stress components along the fault plane are

(C6)
$$\begin{array}{ccc} {\bar{\sigma }}_{n}\left(y\;\cot\;\theta ,y\right) & = & \left(G_{xx}-\pi \delta_{\Omega }\right){\sin\;}^{2}\;\theta +\left(G_{yy}-\pi \delta_{\Omega }\right){\cos\;}^{2}\;\theta -2G_{xy}\;\sin\;\theta \;\cos\;\theta \\ & = & -\frac{\pi }{2}\left[\mathrm{sgn}\left(y+b\right)-\mathrm{sgn}\left(y-a\right)+\mathrm{sgn}\left(y-b\right)-\mathrm{sgn}\left(y+a\right)\right]{\sin\;}^{2}\;\theta \\ & & -\cos\;2\;\theta (a\tan\frac{y+b}{y\;\cot\;\theta +c}-a\tan\frac{y-a}{y\;\cot\;\theta +c}+a\tan\frac{y-b}{y\;\cot\;\theta -d}\\ & & -a\tan\frac{y+a}{y\;\cot\;\theta -d}-\pi \delta_{\Omega })+\frac{\sin\;2\;\theta }{4}\ln\frac{f_{4}(y,b)}{f_{4}(y,a)}\\ & & -\frac{\sin\;2\;\theta }{2}\ln\frac{f_{3}\left(y\;\cot\;\theta +c,y+b\right)f_{3}\left(y\;\cot\;\theta -d,y-b\right)}{f_{3}\left(y\;\cot\;\theta +c,y-a\right)f_{3}\left(y\;\cot\;\theta -d,y+a\right)}-\pi \delta_{\Omega }, \end{array}$$

(C7)
$$\begin{array}{ccc} \bar{\tau }\left(y\;\cot\;\theta ,y\right) & = & \sin\;\theta \;\cos\;\theta \left[\left(G_{xx}-\pi \delta_{\Omega }\right)-\left(G_{yy}-\pi \delta_{\Omega }\right)\right]-G_{xy}\left({\cos\;}^{2}\;\theta -{\sin\;}^{2}\;\theta \right)\\ & = & -\frac{\pi }{4}\left[\mathrm{sgn}\left(y+b\right)-\mathrm{sgn}\left(y-a\right)+\mathrm{sgn}\left(y-b\right)-\mathrm{sgn}\left(y+a\right)\right]\sin\;2\;\theta \\ & & +\sin\;2\;\theta (a\tan\frac{y+b}{y\;\cot\;\theta +c}-a\tan\frac{y-a}{y\;\cot\;\theta +c}+a\tan\frac{y-b}{y\;\cot\;\theta -d}\\ & & -a\tan\frac{y+a}{y\;\cot\;\theta -d}-\pi \delta_{\Omega })-\frac{{\sin\;}^{2}\;\theta }{2}\ln\frac{f_{4}(y,b)}{f_{4}(y,a)}\\ & & -\frac{\cos\;2\;\theta }{2}\ln\frac{f_{3}\left(y\;\cot\;\theta +c,y+b\right)f_{3}\left(y\;\cot\;\theta -d,y-b\right)}{f_{3}\left(y\;\cot\;\theta +c,y-a\right)f_{3}\left(y\;\cot\;\theta -d,y+a\right)}, \end{array}$$

where $G_{ij}\left(y\;\cot\;\theta ,y\right)$ is shortened into $G_{ij}$ for convenience, and the four corners P1, P2, P3, and P4 on the fault plane are singularities (Figure 1). To simplify Equations B18–B20 into Equations C1 and C2, we apply the general rule of taking the right limit, i.e., the limit that the argument approaches the fault from its right‐hand side, as the value of the fault plane. Thus, the segment P1–P2 belongs to the inclusion, while the segment P3–P4 belongs to the surroundings for a permeable fault in the above equations.

In particular, for a vertical fault (θ=90), we obtain

(C8)
$$\begin{array}{ccc} {\bar{\sigma }}_{n}\left(0,y\right) & = & G_{xx}\left(0,y\right)-\pi \delta_{\Omega }\\ & = & \frac{\pi }{2}\left[\mathrm{sgn}\left(y-a\right)-\mathrm{sgn}\left(y+b\right)-\mathrm{sgn}\left(y-b\right)+\mathrm{sgn}\left(y+a\right)\right]\\ & & +a\tan\frac{y+b}{c}-a\tan\frac{y-a}{c}-a\tan\frac{y-b}{d}+a\tan\frac{y+a}{d}-2\pi \delta_{\Omega }, \end{array}$$

(C9)
$$\begin{array}{c} \bar{\tau }\left(0,y\right)=G_{xy}\left(0,y\right)\\ =\frac{1}{2}\ln\frac{f_{3}\left(c,y+b\right)f_{3}\left(d,y-b\right)}{f_{3}\left(c,y-a\right)f_{3}\left(d,y+a\right)}-\frac{1}{2}\ln\frac{f_{4}\left(y,b\right)}{f_{4}\left(y,a\right)}, \end{array}$$

and for a zero offset fault, we have

(C10)
$$\begin{array}{ccc} {\bar{\sigma }}_{n}\left(y\;\cot\;\theta ,y\right) & = & -\cos\;2\;\theta (a\tan\frac{y+b}{y\;\cot\;\theta +c}-a\tan\frac{y-a}{y\;\cot\;\theta +c}\\ & & +a\tan\frac{y-b}{y\;\cot\;\theta -d}-a\tan\frac{y+a}{y\;\cot\;\theta -d}-\pi \delta_{\Omega })\\ & & -\frac{\sin\;2\;\theta }{2}\ln\frac{f_{3}\left(y\;\cot\;\theta +c,y+b\right)f_{3}\left(y\;\cot\;\theta -d,y-b\right)}{f_{3}\left(y\;\cot\;\theta +c,y-a\right)f_{3}\left(y\;\cot\;\theta -d,y+a\right)}-\pi \delta_{\Omega }, \end{array}$$

(C11)
$$\begin{array}{ccc} \bar{\tau }\left(y\;\cot\;\theta ,y\right) & = & \sin\;2\;\theta (a\tan\frac{y+b}{y\;\cot\;\theta +c}-a\tan\frac{y-a}{y\;\cot\;\theta +c}\\ & & +a\tan\frac{y-b}{y\;\cot\;\theta -d}-a\tan\frac{y+a}{y\;\cot\;\theta -d}-\pi \delta_{\Omega })\\ & & -\frac{\cos\;2\;\theta }{2}\ln\frac{f_{3}\left(y\;\cot\;\theta +c,y+b\right)f_{3}\left(y\;\cot\;\theta -d,y-b\right)}{f_{3}\left(y\;\cot\;\theta +c,y-a\right)f_{3}\left(y\;\cot\;\theta -d,y+a\right)}. \end{array}$$
