# Supplementary material for: Analytical Solution to Assess the Induced Seismicity Potential of Faults in Pressurized and Depleted Reservoirs
Source: J Geophys Res Solid Earth. 2021 Jan 19;126(1):e2020JB020436. doi: 10.1029/2020JB020436 (PMC9285632; doi:10.1029/2020JB020436)
Supplement: Supplementary file 1 — Supporting Information S1 [file JGRB-126-0-s001.pdf]

[Journal of Geophysical Research: Solid Earth]

Supporting Information for

[Analytical solution to assess the induced seismicity potential of faults in pressurized and depleted reservoirs]

[Haiqing Wu[1,2], Victor Vilarrasa[2,3,4], Silvia De Simone[5], Maarten Saaltink[1,2] and Francesco Parisio[6]]

[[1]Department of Civil and Environmental Engineering (DECA), Universitat Politécnica de Catalunya (UPC), Jordi Girona 1-3, 08034 Barcelona, Spain
[2]Associated Unit: Hydrogeology Group (UPC-CSIC)
[3]Institute of Environmental Assessment and Water Research (IDAEA), CSIC, c/ Jordi Girona 18, 08034 Barcelona, Spain
[4]Mediterranean Institute for Advanced Studies (IMEDEA), Spanish National Research Council (CSIC), Esporles, Spain
[5]Univ Rennes, CNRS, Géosciences Rennes - UMR 6118, Rennes, France
[6]Chair of Soil Mechanics and Foundation Engineering, Technische Universität Bergakademie Freiberg, Germany]

Contents of this file

Text S1 to S2
Figures S1 to S9
Table S1

Introduction

Three sections are included in the Supporting Information. Section S1 contains the detailed comparison between our analytical solution and the numerical solution of the fully coupled finite element code CODE_BRIGHT (Olivella et al., 1994; 1996) for both permeable and impermeable fault with a dip angle 60°(Text S1 and Figures S1 to S6). Section S2 presents the analytical results of induced stress for a vertical permeable fault (Text S2 and Figures S7 to S9). Finally, in Section S3, the most relevant symbols used in the main text and in this text are listed (Table S1).

Text S1. Detailed comparison between our analytical solution and CODE_BRIGHT for an inclined displaced fault

For verification purposes of the analytical solution, we compare in the main text (Figure 2) the horizontal and vertical total stress as well as the shear stress along the fault resulting from our analytical solution and the numerical results obtained with CODE_BRIGHT. Here, we present a

more detailed comparison of the results, showing the contour plots and the line diagrams on induced horizontal, vertical and shear stress components in Figures S1 to S6. Each figure includes four parts, (a) is the results of the analytical solution developed in the main text, (b) is the simulated results of CODE_BRIGHT, (c) and (d) are the comparisons between the analytical solution and CODE_BRIGHT at the lines x = 0 m, x = 100 m, and y = 0 m, y = 150 m, respectively. To show the features clearly, we plot both the analytical and numerical results focusing on a rectangle of 800 m by 600 m centered in the fault. While Figures S1 to S3 show the results for a permeable fault, Figures S4 to S6 display the results for an impermeable fault. Figures S1-S6 clearly show that the analytical results are almost identical to the numerical results, only the maximum and minimum values have a little difference because the corners are singularities for the analytical solution, which leads to an infinite stress (for representation purposes, the infinite stress is cut off to a finite value), while there is no singularity for numerical solution. Additionally, the discrete nature of the numerical solution also contributes to such discrepancies. Thus, the absolute maximum and minimum values in the numerical results are always smaller than those of the analytical results.

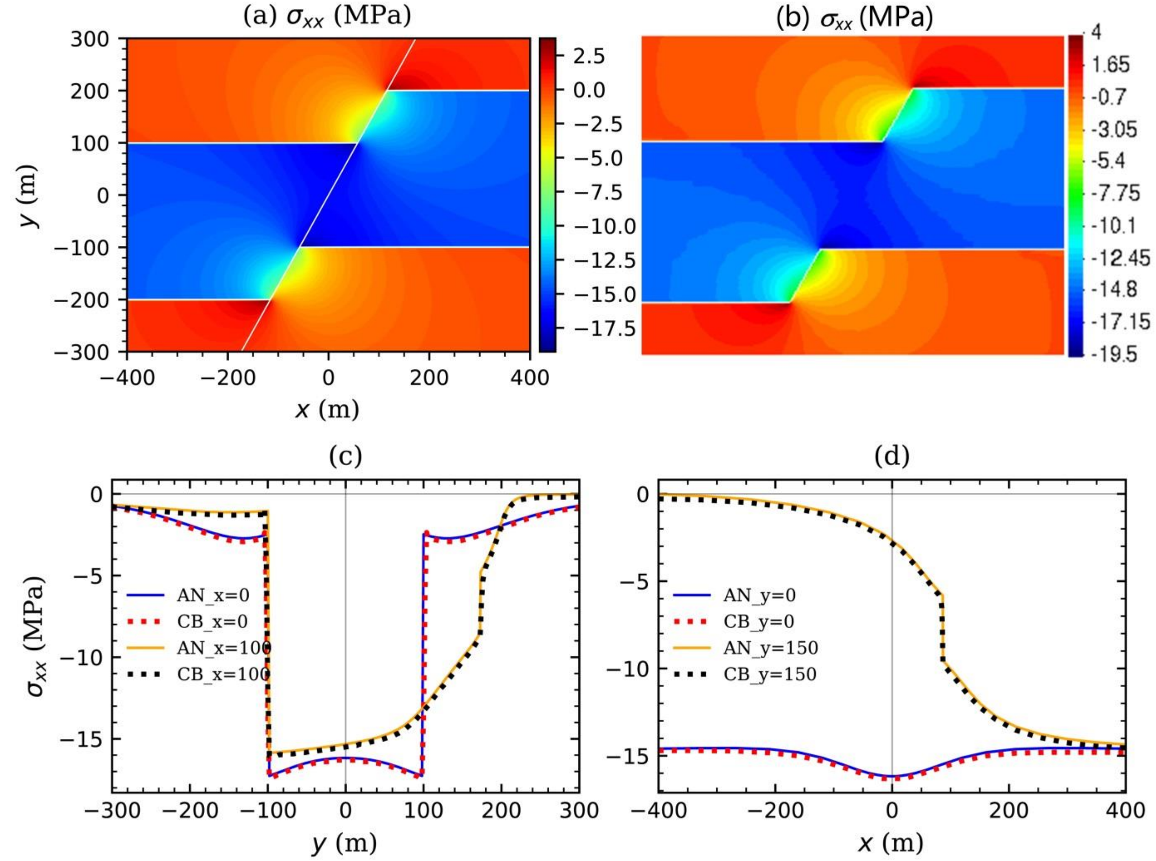

Figure S1. Detailed comparison of the induced horizontal stress for a permeable fault between (a) the analytical solution (AN) and (b) the numerical results of CODE_BRIGHT (CB), as well as the specific comparison of them at the lines (c) x = 0 and x = 100 m, and (d) y = 0 and y = 150 m. The reservoir geometry and the fault are indicated by the white lines in (a).

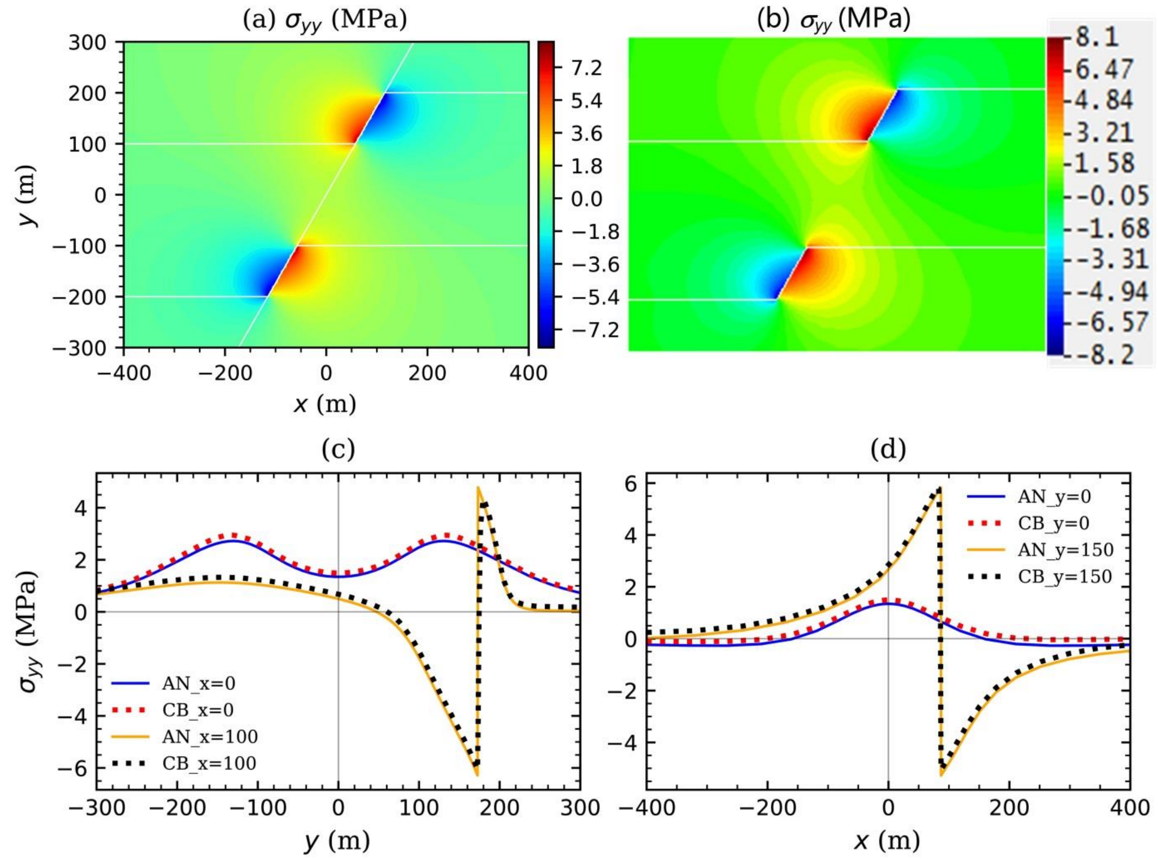

Figure S2. Detailed comparison of the induced vertical stress for a permeable fault between (a) the analytical solution (AN) and (b) the numerical results of CODE_BRIGHT (CB), as well as the specific comparison of them at the lines (c) x = 0 and x = 100 m, and (d) y = 0 and y = 150 m. The reservoir geometry and the fault are indicated by the white lines in (a).

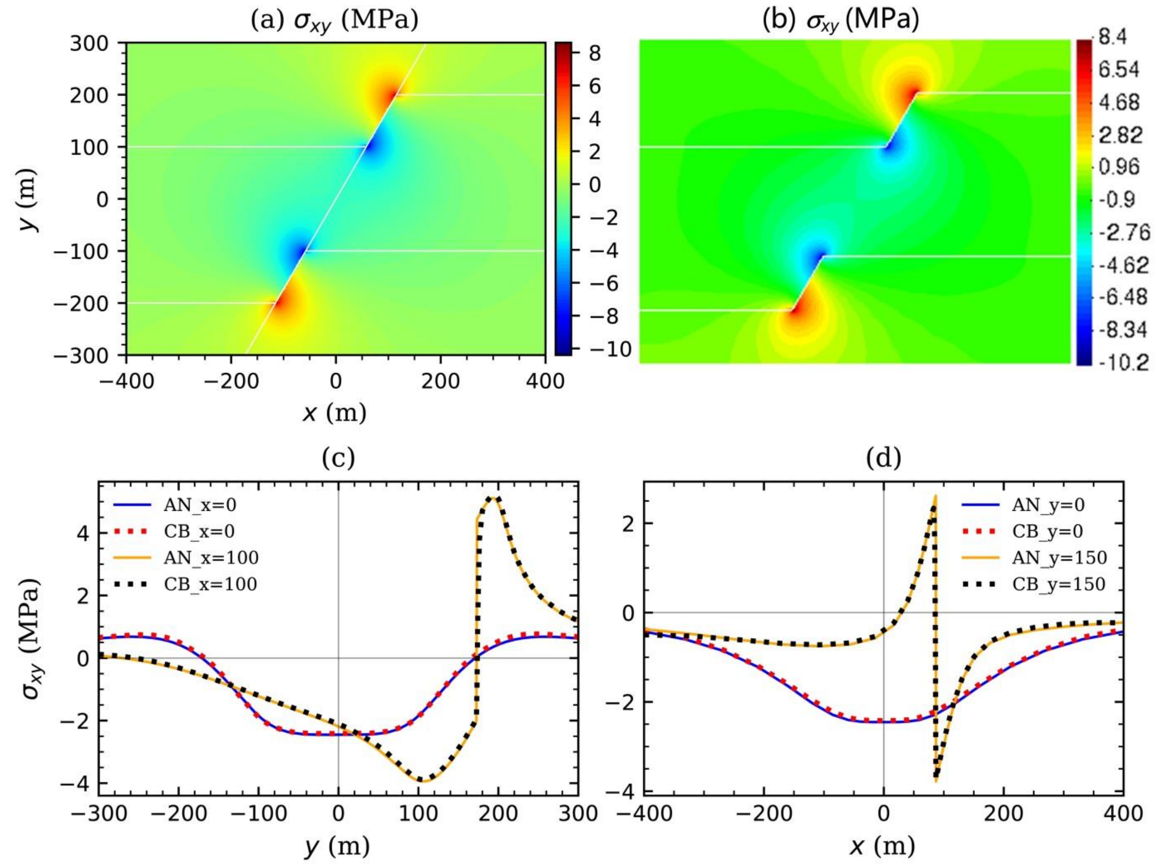

Figure S3. Detailed comparison of the induced shear stress for a permeable fault between (a) the analytical solution (AN) and (b) the numerical results of CODE_BRIGHT (CB), as well as the specific comparison of them at the lines (c) x = 0 and x = 100 m, and (d) y = 0 and y = 150 m. The reservoir geometry and the fault are indicated by the white lines in (a).

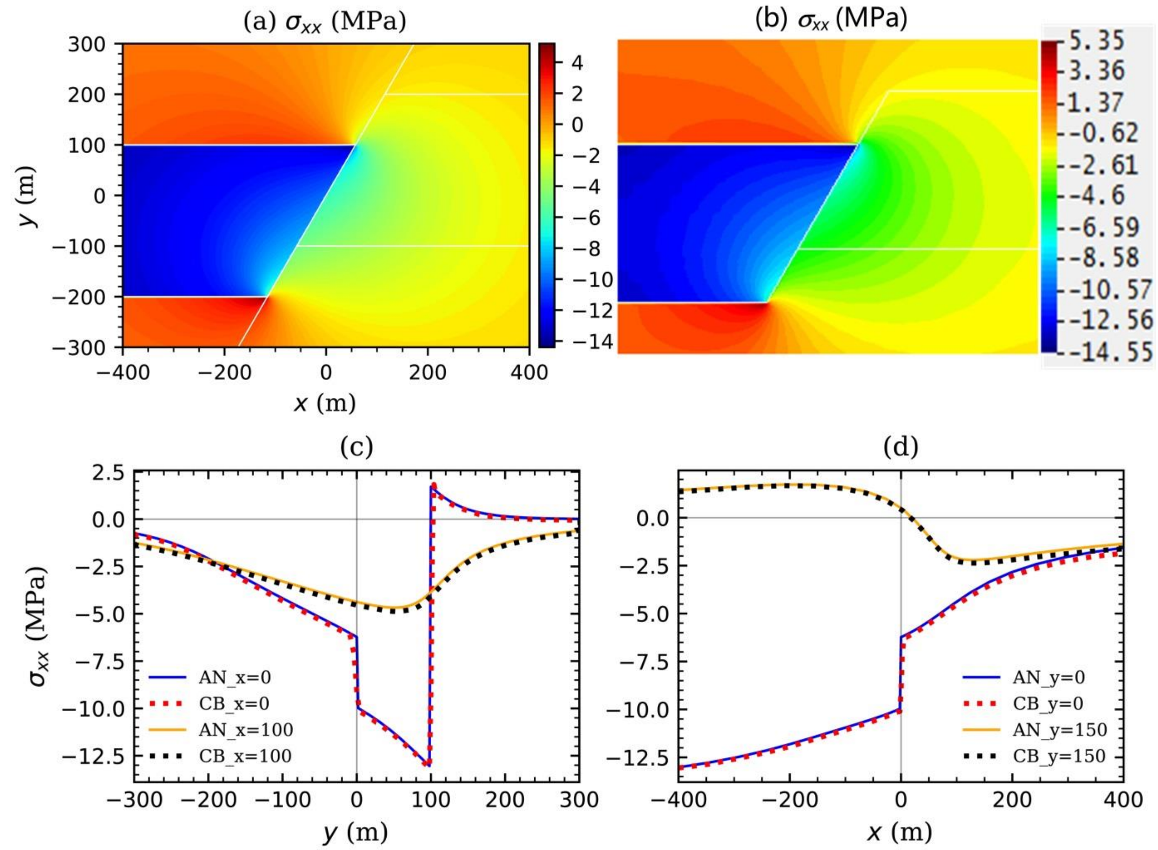

Figure S4. Detailed comparison of the induced horizontal stress for an impermeable fault between (a) the analytical solution (AN) and (b) the numerical results of CODE_BRIGHT (CB), as well as the specific comparison of them at the lines (c) $x = 0$ and $x = 100$ m, and (d) $y = 0$ and $y = 150$ m. The reservoir geometry and the fault are indicated by the white lines in (a).

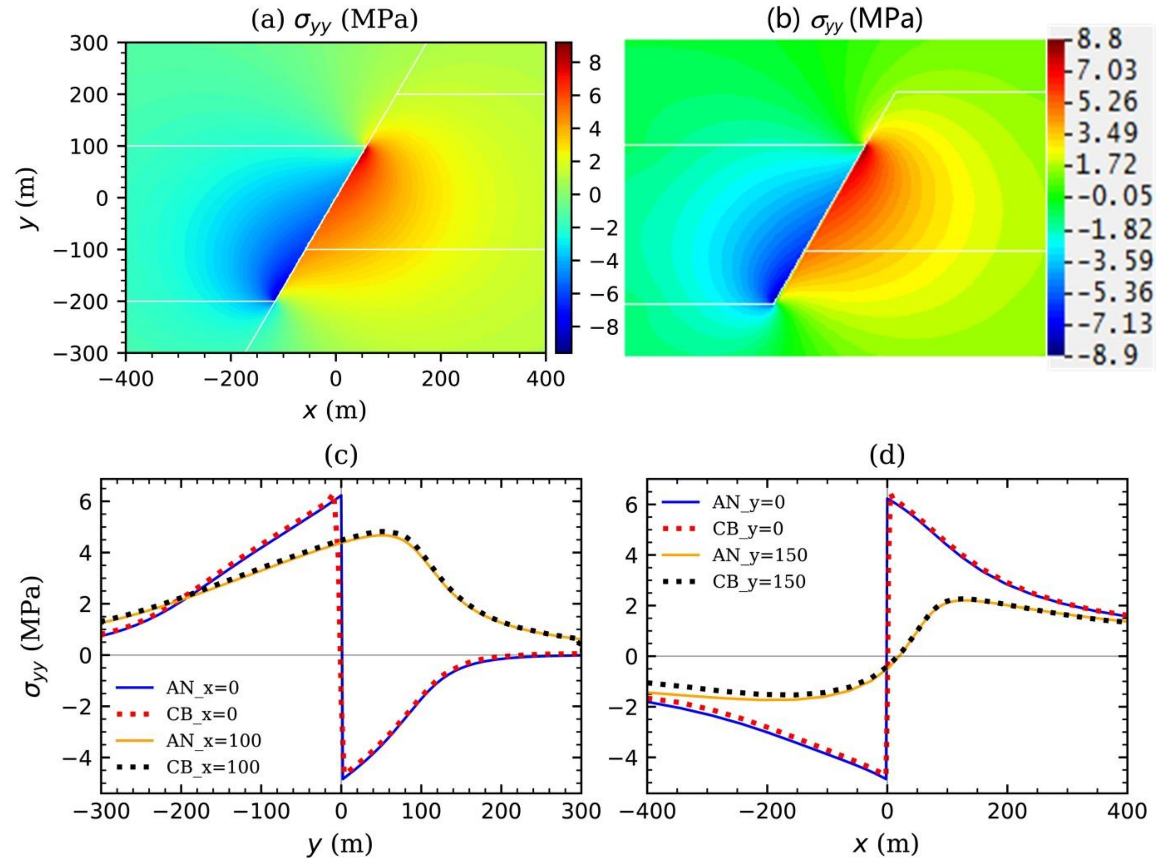

Figure S5. Detailed comparison of the induced vertical stress for an impermeable fault between (a) the analytical solution (AN) and (b) the numerical results of CODE_BRIGHT (CB), as well as the specific comparison of them at the lines (c) x = 0 and x = 100 m, and (d) y = 0 and y = 150 m. The reservoir geometry and the fault are indicated by the white lines in (a).

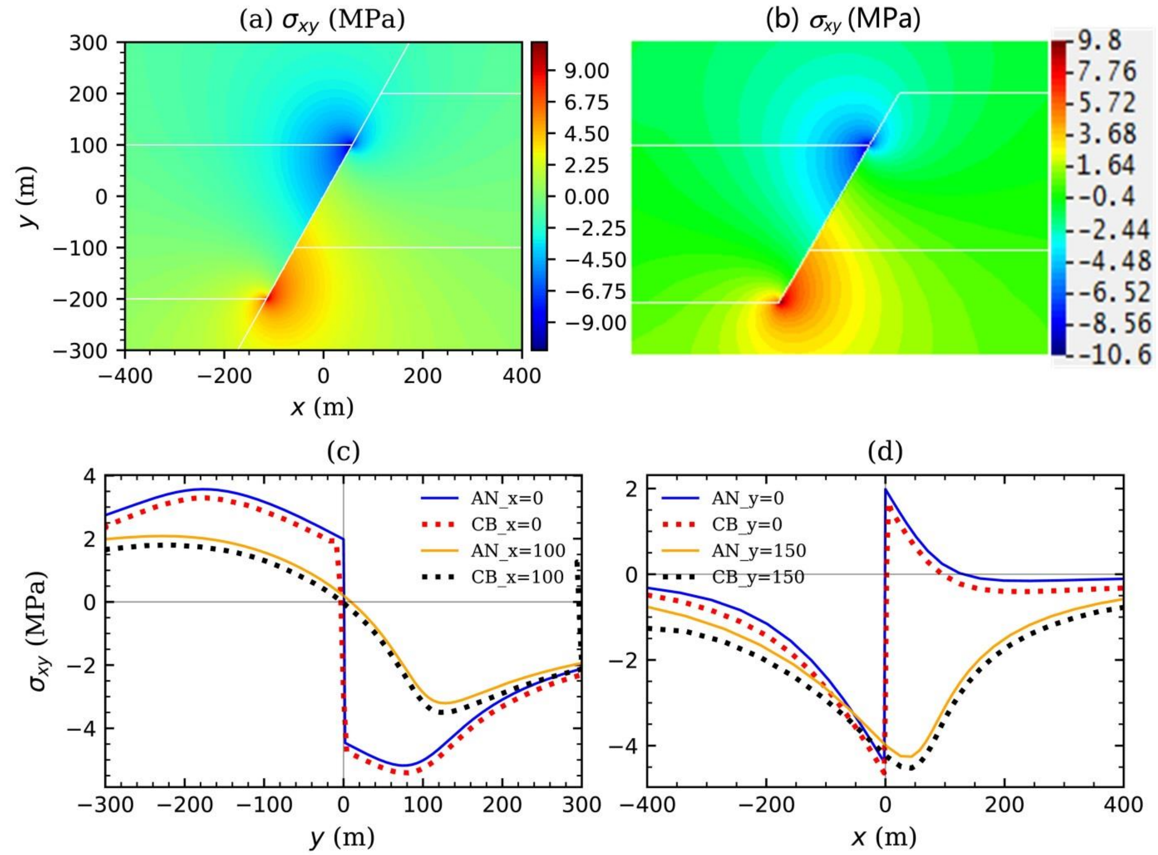

Figure S6. Detailed comparison of the induced shear stress for an impermeable fault between (a) the analytical solution (AN) and (b) the numerical results of CODE_BRIGHT (CB), as well as the specific comparison of them at the lines (c) x = 0 and x = 100 m, and (d) y = 0 and y = 150 m. The reservoir geometry and the fault are indicated by the white lines in (a).

Text S2. Analytical results for a vertical permeable fault

We plot the analytical results for a vertical permeable fault, which aims at comparing our solution with the results of Jansen et al. (2019). Using the same example as Jansen et al. (2019), we find that Figures S7 to S9 in this paper are identical to Figures 6 to 8 in Jansen et al. (2019). Thus, the analytical solution developed in this work is verified again by comparing it with an existing analytical solution.

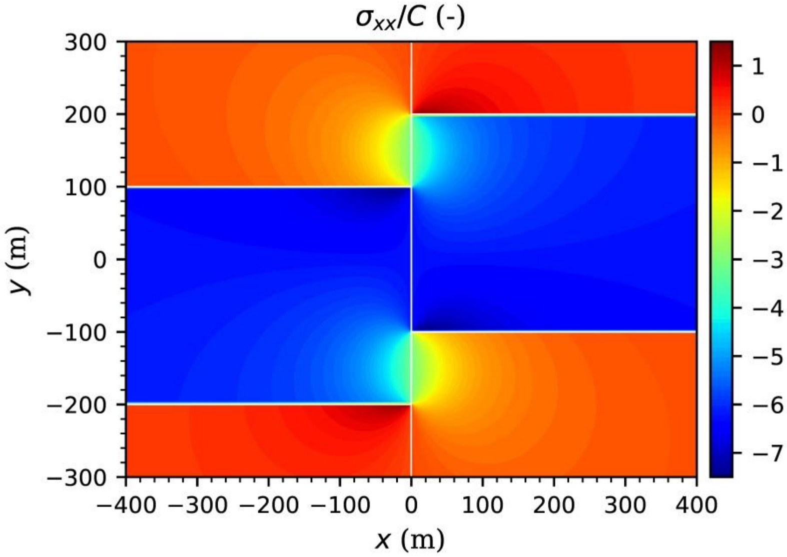

Figure S7. Dimensionless induced horizontal stress for a vertical permeable fault calculated using our analytical solution. The scaling parameter C is 2.36 MPa for the parameter values listed in Table 1. The reservoir geometry and the fault are indicated by the white lines.

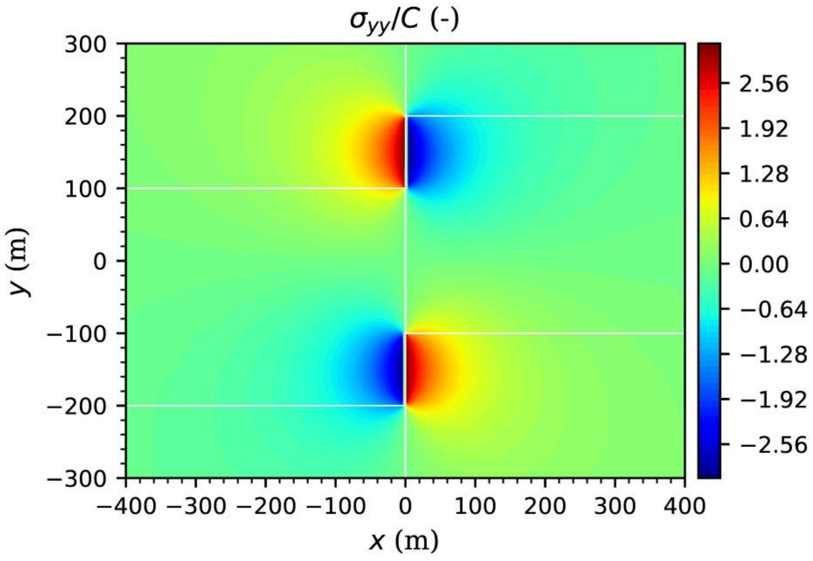

Figure S8. Dimensionless induced vertical stress for a vertical permeable fault calculated using our analytical solution. The scaling parameter C is the same as in Figure S7. The reservoir geometry and the fault are indicated by the white lines.

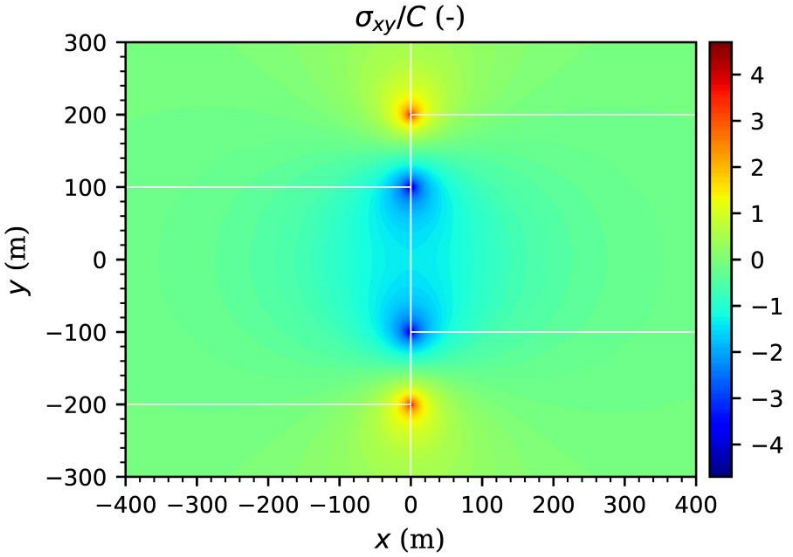

Figure S9. Dimensionless induced shear stress for a vertical permeable fault calculated using our analytical solution. The scaling parameter C is the same as in Figure S7. The reservoir geometry and the fault are indicated by the white lines.

Symbol	Physical meaning	SI unit
x, y	Cartesian coordinates	m
ς, ξ	x and y coordinate values inside inclusion	m
Ω	Inclusion domain	-
a, b, c, d	Geometrical parameters	m
θ	Fault dip	$^\circ$
θ_o	Onset dip angle	$^\circ$
θ_c	Critical dip angle	$^\circ$
ht	Fault offset	m
C	Scaling parameter for stress	N/m^2
D	Scaling parameter for displacement	-
Δp	Pore pressure change	N/m^2
p^0	Initial pore pressure	N/m^2
μ	Shear modulus	N/m^2
K	Bulk modulus	N/m^2
ν	Poisson's ratio	-
α	Biot's coefficient	-
ϵ^*	Eigenstrain	-
ϵ_{kk}	Volumetric strain	-
ϵ_{ij}	Induced strain	-
g_i	Green's function for displacement	1/m
g_{ij}	Green's function for stress	$1/m^2$
G_{ij}	Surface integral of the Green's function g_{ij}	-
R	Radial distance from nucleus of strain	m
u_i	Displacement	m

σ^*	Eigenstress	N/m^2
σ_{ij}	Induced stress	N/m^2
τ	Shear stress along the fault plane	N/m^2
σ_n'	Effective normal stress along the fault plane	N/m^2
$\overline{\tau}$	Dimensionless induced shear stress along the fault plane	-
$\overline{\sigma}_n$	Dimensionless induced normal stress along the fault plane	-
σ_{ij}^0	Initial stress	N/m^2
k_0	Stress ratio of horizontal to vertical stress	-
k_0^c	Critical stress ratio	-
η_{st}	Static friction coefficient	-
CFS	Coulomb failure stress	N/m^2
ΔCFS	Coulomb failure stress change	N/m^2
L_s	Characteristic length of fault	m
ℓ_i	A continuous interval in coordinate y	m
S_i	Fault slip size	m
$S_{\max}$	The maximum fault slip size	m
Subscript D	Dimensionless variables	-

Table S1. Instruction of symbols used in this study.

References

Jansen, J. D., Singhal, P., & Vossepoel, F. C. (2019). Insights from closed - form expressions for injection - and production - induced stresses in displaced faults. Journal of Geophysical Research: Solid Earth. 124(7), 7193-7212. https://doi.org/ 10.1029/2019JB017932

Olivella, S., Carrera, J., Gens, A., & Alonso, E. E. (1994). Nonisothermal multiphase flow of brine and gas through saline media. Transport in porous media, 15(3), 271-293.

Olivella, S., Gens, A., Carrera, J., & Alonso, E. E. (1996). Numerical formulation for a simulator (CODE_BRIGHT) for the coupled analysis of saline media. Engineering computations, 13(7), 87-112.
